# Mitochondrial-Nuclear DNA Interactions Contribute to the Regulation of Nuclear Transcript Levels as Part of the Inter-Organelle Communication System

**DOI:** 10.1371/journal.pone.0030943

**Published:** 2012-01-23

**Authors:** Chris D. M. Rodley, Ralph S. Grand, Lutz R. Gehlen, Gary Greyling, M. Beatrix Jones, Justin M. O'Sullivan

**Affiliations:** 1 Institute of Natural Sciences, Massey University, Albany, New Zealand; 2 Centre for Mathematical Biology, Massey University, Albany, New Zealand; Lehigh University, United States of America

## Abstract

Nuclear and mitochondrial organelles must maintain a communication system. Loci on the mitochondrial genome were recently reported to interact with nuclear loci. To determine whether this is part of a DNA based communication system we used genome conformation capture to map the global network of DNA-DNA interactions between the mitochondrial and nuclear genomes (Mito-nDNA) in *Saccharomyces cerevisiae* cells grown under three different metabolic conditions. The interactions that form between mitochondrial and nuclear loci are dependent on the metabolic state of the yeast. Moreover, the frequency of specific mitochondrial - nuclear interactions (*i.e. COX1*-*MSY1* and *Q0182*-*RSM7*) showed significant reductions in the absence of mitochondrial encoded reverse transcriptase machinery. Furthermore, these reductions correlated with increases in the transcript levels of the nuclear loci (*MSY1* and *RSM7*). We propose that these interactions represent an inter-organelle DNA mediated communication system and that reverse transcription of mitochondrial RNA plays a role in this process.

## Introduction

Mitochondria have a central role within the metabolic systems of cells. In yeast (*Saccharomyces cerevisiae*), as in other organisms, the mitochondrial organelle contains a genome that encodes an essential subset of the electron transport chain components [1] that are necessary for respiratory growth [2].

The mitochondrial genome has drastically reduced in size during the course of evolution to the point that ∼98% of the genes required for mitochondrial function are encoded within the nuclear chromosomes [3]. Consequently, mechanisms must exist to co-ordinate and control the expression of the nuclear- and mitochondrial genome- encoded genes in order to maintain and control mitochondrial function [4], [5]. Intriguingly, despite the fact that the majority of mitochondrial genes have transferred to the nuclear genome, transfer of mitochondrial DNA (mtDNA) to the yeast nucleus remains an on-going process with mtDNA being used to repair double stranded breaks in yeast nuclear chromosomes under certain conditions [6]. Unstable mitochondrial plasmids have also been observed to transfer into the yeast nucleus [7], [8] in a nuclear gene (*e.g. YME1*, *YME2*) dependent manner [8]–[10]. The nuclear functions of these transferred mtDNAs are unknown, however elevated mitochondria to nucleus DNA migration rates correlate with accelerated chronological aging in yeast [11].

Distal regulatory regions (*e.g.* enhancers) are known to loop within chromosomes in order to interact with the promoter region of the genes that they control [12]. Furthermore, enhancers can also interact with promoters on different chromosomes to control gene expression [13], [14]. These types of inter- and intra-chromosomal interactions can be captured using proximity-based ligation methodologies (*e.g.* Chromosome Conformation Capture (3C) [15]) that incorporate high resolution (*i.e.* ∼2 Å [16]) cross-linking of interacting DNA strands, restriction digestion, dilution, and ligation to identify DNA sequences that interact within a cell. Using a proximity-based ligation method that we developed to observe the global set of genome wide interactions (Genome Conformation Capture (GCC)), we previously observed that nucleic acids of mitochondrial origin interact with nuclear loci (hereinafter referred to as Mito-nDNA interactions) in *S. cerevisiae* [17]. Surprisingly these inter-organelle, Mito-nDNA interactions are frequent and statistically significant suggesting that they perform a hitherto unrecognized role within yeast cells [17]. Furthermore, analysis of one of these interactions demonstrated carbon source dependence [17]. Intriguingly, the quality and quantity of mitochondrial DNA has been shown to affect patterns of nuclear transcription [18], [19] and replication [20] in yeast.

In this study we explore the hypothesis that inter-organelle interactions respond to the metabolic status of the cell to regulate nuclear transcript levels. Using GCC we identify dramatic differences in both the frequency and identities of inter-organelle interactions occurring in *S. cerevisiae* during growth on glucose, galactose (*i.e.* respiro-fermentation [21], [22]), and glycerol lactate (*i.e.* solely respiration). We also demonstrate that Interactions between mitochondrial genes (*i.e. COX1* and *Q0182*, a dubious mitochondrial ORF) and nuclear encoded loci (*i.e. MSY1* and, *RSM7*, respectively), are dependent upon a functional electron transport chain and mitochondrial encoded reverse transcriptase machinery. Finally, the levels of the nuclear encoded *MSY1* and *RSM7* gene transcripts are increased when the interaction frequency is reduced by the knockout of mitochondrial reverse transcriptase activity. On the basis of these results we propose that reverse-transcription mediated inter-organelle DNA interactions are a novel form of communication between mitochondria and the nucleus.

## Results

We previously captured Mito-nDNA interactions in *S. cerevisiae* cells grown in glucose by GCC [17]. A detailed investigation of one of these Mito-nDNA interactions (between the *COX1* gene (Mt: 24872–26193 bp) and the nuclear encoded *MSY1* gene (Chr XVI; 365496–365760 bp), herein after denoted *COX1-MSY*) [17] demonstrated that it was carbon source dependent. Therefore, we hypothesized that Mito-nDNA interactions would alter, on a global scale, according to the cell's metabolic state, and in particular, the carbon source used for growth. Thus, we used GCC to generate comprehensive maps of the Mito-nDNA interactions in *S. cerevisiae* during exponential growth in media containing glucose, galactose, or glycerol lactate. Two biological replicates were prepared and analyzed for each condition. Interaction networks were constructed from 36 bp paired end Illumina Genome Analyzer sequence reads (total reads; glucose 56,167,792, galactose 48,419,385, glycerol lactate 49,134,906) of GCC libraries prepared using *MspI* digested chromatin.

Statistical and experimental methods were used to determine if the Mito-nDNA interaction patterns could have been generated by experimental noise alone, which would be expected to produce random pairings of fragments from the two genomes. *In silico* simulations (100,000) were performed [17] to determine the maximum count of a particular interaction that would be observed under this random noise model, given the same number of sequences, interactions and fragments as in the experimental data. These results showed that the real dataset deviates from a random distribution and, therefore, we conclude that the interaction patterns cannot be attributed to noise alone under any of the conditions, in each case with a p-value less than 10^−5^. Subsequently, we performed analyses to determine what frequency individual interactions have to achieve before they are deemed to be present at a level above experimental noise (Methods S1). As a result, we identified 8678 statistically significant interactions occurring between the nuclear and mitochondrial genomes during glucose growth, 1780 during galactose growth, and 8153 during growth in glycerol lactate (Table 1). The numbers of interactions in each condition did not correlate with the measured mitochondrial copy number (Table S1). Biological replicates for each condition were highly correlated for statistically significant interactions (R^2^ = 0.78, 0.93, 0.93, respectively; Figure S1 and Methods S1). Accordingly, sequences from biological replicates were combined and reanalyzed.

**Table 1:** Inter-organelle interactions are carbon source dependent.

	Glucose	Glycerol Lactate	Galactose
**Mito-nDNA Interactions**	363	3879	278
**Mito-rDNA Interactions**	8315	4274	1512
**Total**	**8678**	**8153**	**1780**

There was a >10 fold increase in the number of Mito-nDNA interactions during growth in glycerol lactate (respiration) as compared to growth in glucose and galactose (respiro-fermentation). Growth on galactose resulted in less Mito-nDNA and Mito-rDNA interactions combined, compared to the other two conditions. Statistically significant DNA-DNA interactions were divided according to whether the mtDNA was interacting with nuclear rDNA, or with unique nuclear loci. Corrections for the copy numbers of the rDNA repeats and the mitochondrial genome were incorporated into the significance calculations (Methods S1).

To experimentally control for spurious inter-molecular ligation events during the GCC process, samples were spiked with two ligation controls during library preparation. The first ligation control consisted of PCR products amplified from *Escherichia coli* or Lambda bacteriophage (Table S2) that were added (1∶1 ratio with the nuclear genome copy number) before the GCC ligation step. These controls were designed to estimate the frequency of random inter-molecular ligation events during GCC library preparation. A maximum of 47 separate ligation events were observed, none of which occurred at levels above the statistically defined experimental noise threshold. The second ligation control consisted of the addition of pUC19 plasmid to the sample following the GCC ligation in order to control for random ligation events during high-throughput sequencing library preparation. We observed a maximum of six interactions between pUC19 and the rest of the genome; again none of these interactions were above the statistically defined experimental noise threshold. These controls show that random inter-molecular ligations occur at very low frequencies that are below our noise threshold for significant interactions. This is true even for the high copy number rDNA and mitochondrial DNA elements and thus provides empirical evidence that random ligation events during sample preparation do not account for the interactions we observe.

Significant interactions were separated into two pools, those which occur between the mtDNA and the nuclear ribosomal DNA repeats (Mito-rDNA), and those between mtDNA and unique nuclear loci (Mito-nDNA; Table 1). The rDNA repeats form part of the nucleolus and encode the rRNA component of the cytosolic ribosomes. The rDNA repeats constitute ∼9.8% of the yeast genome; yet, the Mito-rDNA interactions constitute 95.8%, 52.4%, and 84.5% of the total interactions between the nuclear and mitochondrial genomes in glucose, glycerol lactate, and galactose, grown cells, respectively. There does not appear to be an interaction “hotspot”, with Mito-rDNA interactions evenly spread across the 9.1 kb rDNA repeat (data not shown). Hence, Mito-rDNA interactions are over-represented within the data-set and are carbon source dependent (Table 1). We also observed considerable alterations to the numbers of Mito-nDNA interactions. Moreover, the mitochondrial regions that are involved in interactions with the nDNA are not uniformly distributed across the mitochondrial genome (Figure S2).

**Figure 1:**
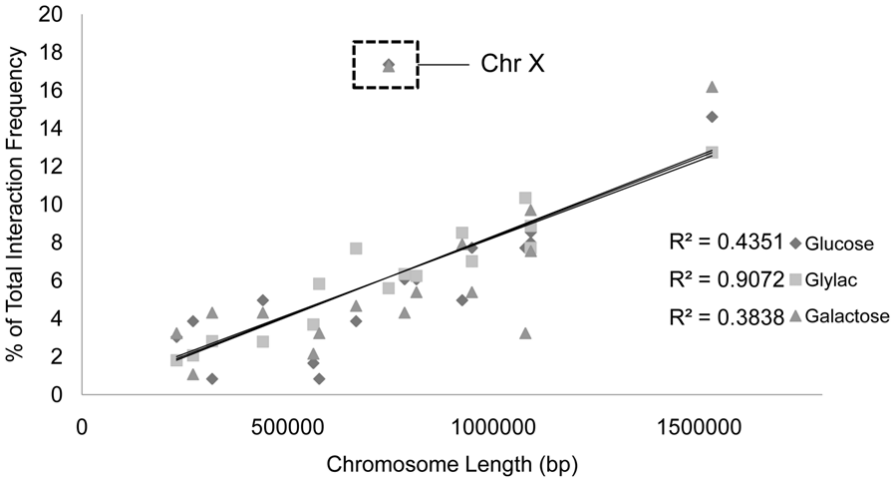
The number of Mito-nDNA interactions correlates with chromosome length, except chromosome X. Statistically significant Mito-nDNA interactions, occurring above the expected noise level (selected to have a false positive rate of between 1 and 3%), have been summed for each nuclear chromosome and expressed as a percentage of the total number of interactions for the particular sample before being plotted according to chromosome length in base pairs. Interactions included in this analysis are between the mitochondrial genome and nuclear chromosomes, with the 2-micron plasmid and rDNA interactions removed. The length of chromosome XII has been reduced to account for the rDNA interactions being removed.

In order to determine whether the observed changes in Mito-nDNA interactions are chromosome specific, we asked whether nuclear chromosome length correlates with the number of interactions identified for each individual chromosome. The number of Mito-nDNA interactions per nuclear chromosome is highly correlated with chromosome length in the glycerol lactate condition, but not in glucose or galactose (Figure 1). This discrepancy is mainly due to the deviation of chromosome X from the trend during growth in glucose and galactose. Intriguingly, the increase in mtDNA interactions with chromosome X is accounted for by a single nuclear *MspI* fragment that encompasses the promoter region and part of the coding sequences of two divergent ORFs: one uncharacterized ORF (YJR115W), and *RSM7* which encodes a mitochondrial small subunit ribosomal protein. Numerous mtDNA *MspI* fragments, including fragments surrounding or overlapping the *COX1*, *COX3*, *VAR1* and *SCE1* genes, interact with this one region on chromosome X.

**Figure 2:**
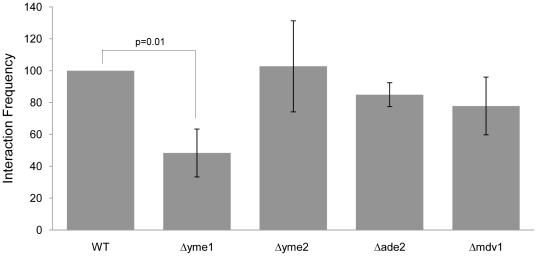
Deletion of *yme1* causes a significant reduction in the frequency of the mitochondrial-nuclear *COX1*-*MSY1* interaction. Interaction frequency between the mitochondrial *COX1* and nuclear *Msp*I fragments was assayed by quantitative 3 C (Methods S1) in wild-type (*S. cerevisiae* BY4741), Δ*yme1* (BY4741 Δ*yme1*), Δ*yme2* (BY4741 Δ*yme2*), Δ*ade2* (BY4741 Δ*ade2*) and Δ*mdv1* (BY4741, Δ*mdv1*) strains. Interaction values were corrected for mitochondrial genome copy number (see Methods) and are expressed as percentages of wild-type (set at 100%) +/− standard error of the mean (n = 4). Deletion of an unconnected gene (*ade2*) did not significantly affect interaction frequency. T-tests (paired P(T< = t) one-tail, n = 4) were performed to determine the significance of observed variations: wild-type: Δ*yme1* p = 0.01; wild-type: Δ*yme2* p = 0.377; wild-type: Δ*ade2* p = 0.103; wild-type: Δ*mdv1* p = 0.143; Δ*yme1*: Δ*mdv1* p = 0.210. Only Δ*yme1* demonstrated a significant difference.

Yeast mitochondrial escape mutants (YME) [8] have been previously implicated in an elevated rate of transfer of unstable mitochondrial plasmids to the yeast nucleus [7]–[10]. Therefore, we predicted that the YME pathway was the source of mtDNA fragments interacting with the nuclear genome, and that mutations within this pathway would result in an increase in the frequency of inter-organelle DNA interactions. To test this prediction, we used quantitative 3 C to compare the frequency of the strong *COX1-MSY1* interaction (identified in [17]) in *S. cerevisiae* YME knockout mutants (*i.e. Δyme1*, *Δyme2*). Contrary to expectations, we observed a significant decrease in the frequency of the *COX1-MSY1* interaction in the Δ*yme1* strain as compared to the wild-type (T-test [Paired P(T< = t) one-tail, n = 4] p = 0.010; Figure 2). Deletion of *YME2* or a functionally unconnected nuclear gene (*ADE2*) did not significantly affect the *COX1-MSY1* interaction frequency (t-test [paired P(T< = t) one-tail, n = 4] p = 0.377 and 0.103 respectively; Figure 2). These results suggest that the source of the mtDNA that participates in the Mito-nDNA interactions is not the unstable mitochondrial plasmids that were previously identified as escaping the mitochondria for the nuclear compartment.

Deletion of *YME1* results in an elevated rate of mitochondrial turn-over as well as an abnormal globular mitochondrial morphology [9], [23]. Therefore, it is possible that this fragmented mitochondrial phenotype contributes to the reduction in the *COX1-MSY1* interaction frequency we observed in the *yme1* deletion strain. To test this we arrested yeast cells with α-factor, which results in a fragmented mitochondrial network [24] that is phenotypically similar to the one observed in *yme1* deletion strains [23]. We also deleted the mitochondrial fission gene (*MDV1*) to create strains that are unable to correctly fragment mitochondria [25], [26]. Interestingly, we observed a similar reduction in the *COX1*-*MSY1* interaction frequency upon α-factor induced synchronization (Figure S3). However, the interaction frequency measured in the Δ*mdv1* strain was intermediate between that observed for the wild-type and Δ*yme1* strains, and not significantly different from either (T-test [Paired P(T< = t) one-tail, n = 4] wt-Δ*mdv1* p = 0.143, Δ*mdv1*-Δ*yme1* p = 0.210; Figure 2). Therefore, it is unlikely that mitochondrial fragmentation is directly responsible for the observed changes in *COX1*-*MSY1* interaction frequency.

**Figure 3:**
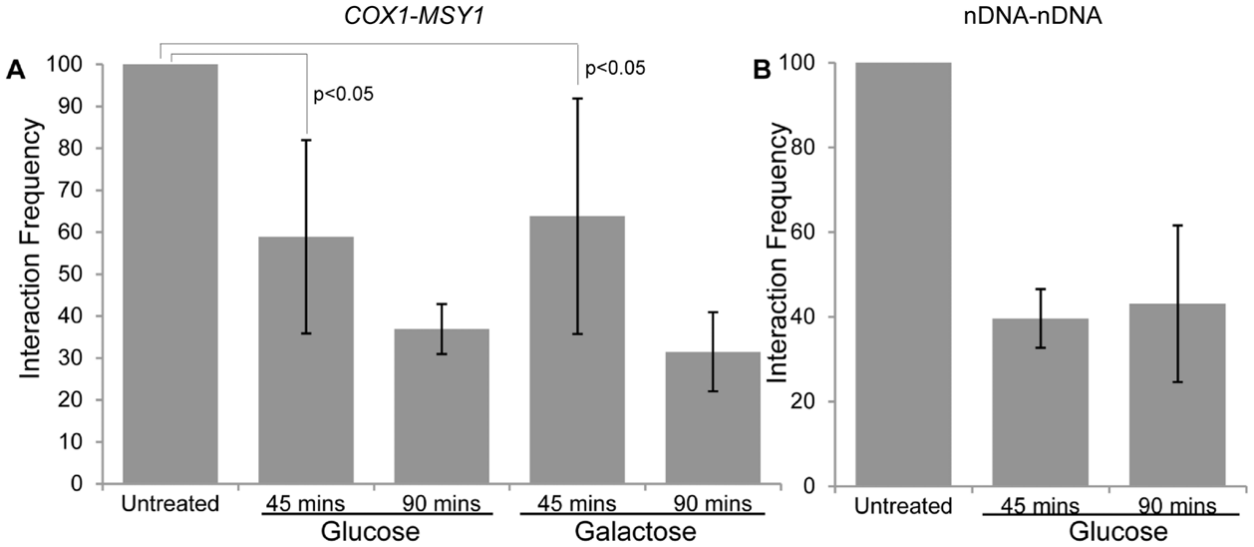
A functional electron transport chain is required to maintain the interaction between the mitochondrial *COX1* and nuclear *MSY1* loci. Uncoupling of the electron transport chain was achieved by 2,4-dinitrophenol (5 mM) treatment of exponentially growing *S. cerevisiae* in synthetic complete media, containing glucose or galactose, for the indicated time (Figure S4). *COX1-MSY1* A) and nDNA-nDNA B) interaction frequencies were determined by quantitative 3 C analyses using fluorescent probes (see Methods S1). Interaction values in A) were corrected for mitochondrial genome copy number while those in B) were corrected for nuclear genome copy number (see Methods). Interaction values were expressed as percentages of the untreated sample (set at 100%) +/− standard error of the mean (n = 3).

We next postulated that an abnormal mitochondrial morphology, coupled with elevated mitochondrial turnover would result in a disturbance of the mitochondrial ATP synthesis pathway, and this may explain the observed reduction in the frequency of the *COX1-MSY1* interaction. Therefore, we tested the inter-organelle *COX1-MSY1* interaction for ATP dependence by treating yeast cells with an electron transport chain uncoupling agent, 2,4-Dinitrophenol (DNP), at a concentration (5 mM) that inhibits respiration but allows fermentation (Figure S4). We observed a significant time-dependent decrease in the frequency of the *COX1-MSY1* interaction in the presence of DNP (t-test p<0.05; Figure 3A), as measured by quantitative 3 C. However, an interaction between two nuclear loci (nDNA-nDNA; Chr VII: 868673–873686 bp - Chr IX: 172565–173311 bp) was also shown to be affected by treatment with DNP (Figure 3B). The observed dependence of the nDNA-nDNA interaction on a proton gradient across the mitochondrial membrane, and thus mitochondrial ATP synthesis, suggests that formation of these DNA-DNA interactions is ATP dependent.

**Figure 4:**
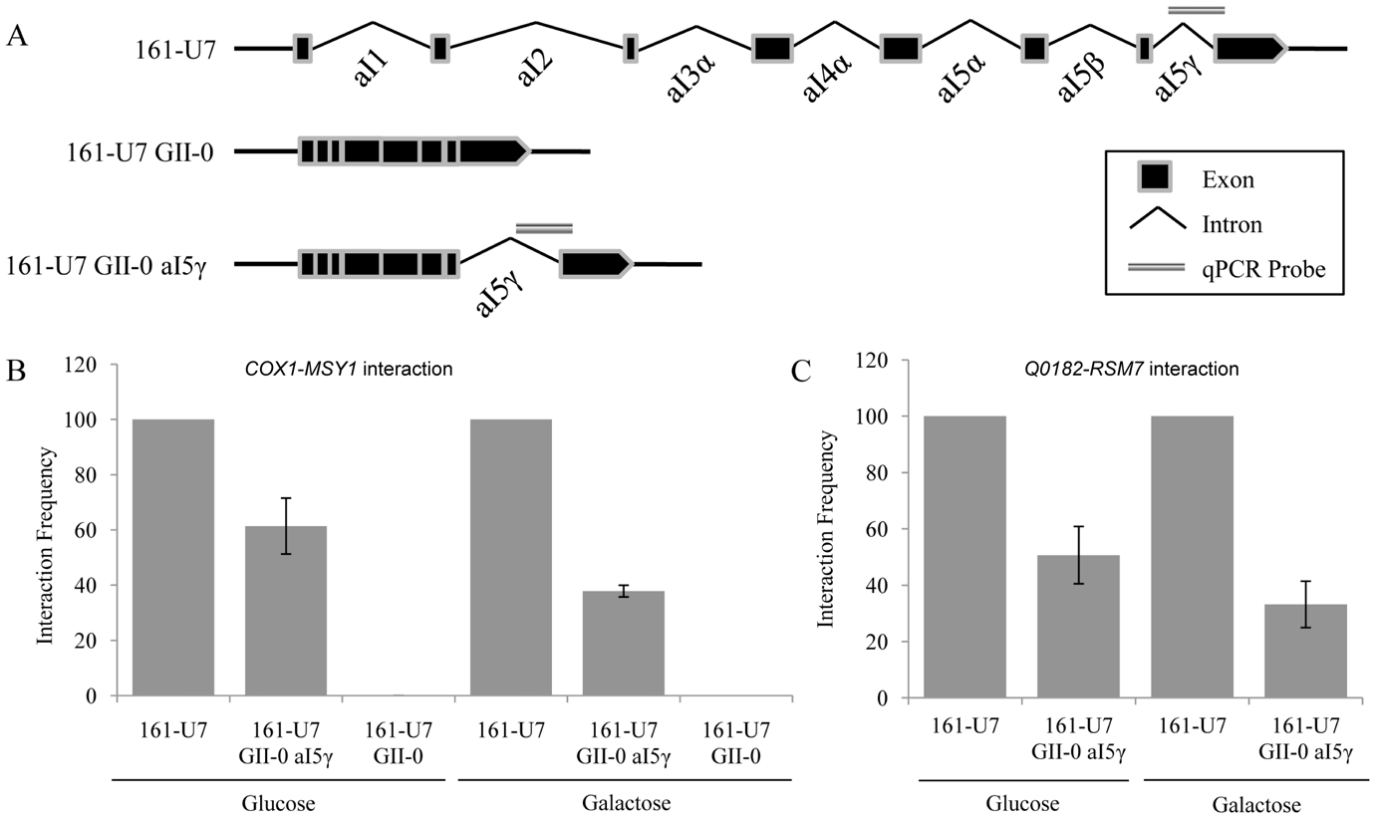
Mito-nDNA interactions require active mitochondrial reverse transcriptase machinery. A) Illustration of *COX1* gene arrangement in the WT (161-U7), intron a15γ (161-U7 GII-0 a15γ), and no mitochondrial group II introns (161-U7 GII-0) strains. Group II introns within the *COX1* gene encode functional reverse transcriptase. The region of *COX1* that participates in the *COX1*-*MSY1* interaction is indicated (qPCR probe). Strain 161-U7 GII-0 was included as a control to rule out a nuclear sequence, originating from a mitochondrial integration within the nuclear genome (NUMT), being responsible for the observed interaction. B) *COX1-MSY1* interaction frequencies for wt and intron mutants, illustrated in A), grown in glucose or galactose. C) *Q0182*-*RSM7* interaction frequencies for mitochondrial reverse transcriptase mutant 161-U7 GII-0 a15γ, illustrated in A), grown in glucose or galactose. Interaction frequencies are expressed as percentages of the wild-type *S. cerevisiae* strain 161-U7 for each carbon source (set at 100%) +/− standard error of the mean (n = 3). Interaction values in B) and C) were corrected for nuclear genome copy number to facilitate direct comparison.

The region of the *COX1* gene involved in the *COX1*-*MSY1* interaction overlaps the non-essential group II mitochondrial aI5γ intron. There are four Group II introns present within yeast mitochondria (aI1, aI2, bI1, and aI5γ) and these introns encode functional splicing, reverse transcriptase, or endonuclease machinery [27]–[31]. Only the aI1 and aI2 introns encode reverse transcriptase [32] activity while the aI5γ intron encodes endounuclease activity [33], [34] but not reverse transcriptase activity [35]. Therefore, we postulated that reverse transcription of the mitochondrial group II introns might be involved in the *COX1*-*MSY1* interaction. To test this we measured the *COX1*-*MSY1* interaction using quantitative 3 C on a strain which only contained the *COX1* aI5γ intron (*i.e.* 161-U7 GII-0 aI5γ; Figure 4A). We observed a 40–60% decrease in the inter-organelle *COX1-MSY1* interaction in the GII-0 aI5γ strain relative to the wild type (Figure 4B). We concluded that the partial dependence of the *COX1*-*MSY1* interaction upon the presence of the group II introns reflects a role for reverse transcription in the inter-organelle interactions. However, part of the *COX1-MSY1* interaction remains independent of mitochondrial encoded reverse transcriptase.

It remained to be seen if interactions involving other mitochondrial loci required the presence of the type II introns and hence reverse transcriptase. We examined the interaction frequency between a dubious mitochondrial ORF (*Q0182*; mtDNA [65783–65903 bp]), that does not contain any group II introns and therefore is not altered in the 161-U7 GII-0 strain, and the nuclear encoded *RSM7* (Chr X [638756–640423 bp]) gene. The *Q0182*-*RMS7* interaction frequency decreased in the absence of the mitochondrial group II introns (Figure 4C). These results confirm that this phenomenon is not restricted to interactions involving *COX1*.

Our results suggested that the nucleic acids of mitochondrial origin which participate in the Mito-nDNA interaction are reverse transcribed from RNA intermediates prior to transfer to the nucleus as cDNAs. However, it remained possible that the Mito-nDNA interactions we observed did not involve inter-organelle transfer. Rather, these interactions might have been completely or partially due to interactions between nuclear loci and mitochondrial sequences that had been integrated into the nuclear genome (*i.e.* nuclear-mitochondrial sequences (NUMTs)). To rule out the possibility that NUMTs were involved, we performed quantitative 3 C analyses in strains without (*i.e.* 161-U7 GII-0) the *COX1* aI5γ intron (Figure 4A and B). Removal of aI5γ, and hence the probe site (Figure 4A), resulted in complete loss of detectable *COX1*-*MSY1* interactions (Figure 4B). This confirmed that the *COX1-MSY1* interaction involves DNA that is directly derived from the mitochondrial genome and not a NUMT.

**Figure 5:**
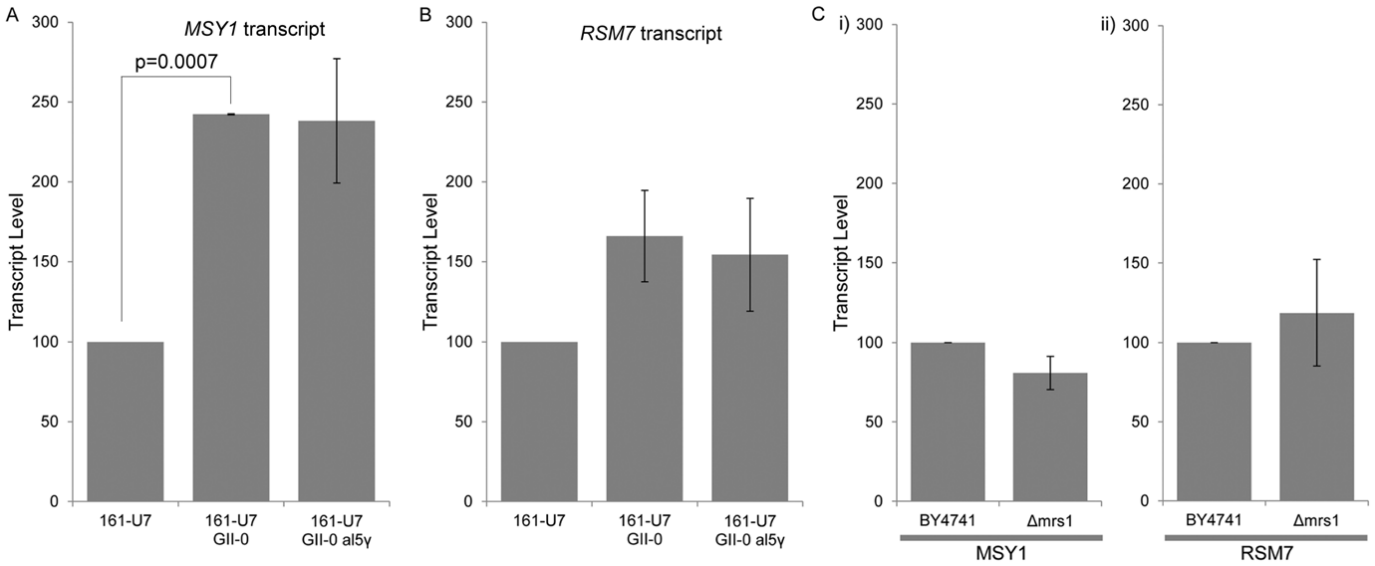
Knocking out mitochondrial encoded reverse transcriptase activity results in increased transcript levels of nuclear genes that are involved in Mito-nDNA interactions. A) Nuclear encoded *MSY1* transcript levels were determined by qRT-PCR in WT (strain 161-U7), 161-U7 GII-0 (lacks both the mitochondrial group II introns and the *COX1* interacting region; Figure 4A), and 161-U7 GII-0 a15γ (contains the interacting region and lacks the group II introns; Figure 4A) cells. B) Nuclear encoded *RSM7* transcript levels were determined by qRT-PCR in: WT (strain 161-U7); 161-U7 GII-0; and 161-U7 GII-0 a15γ cells. Neither 161-U7 GII-0 nor 161-U7 GII-0 a15γ has any alteration within the *Q0182* open reading frame. C) Deletion of *MRS1* (BY4741 Δ*mrs1*), a nuclear gene involved in splicing mitochondrial type-I introns, has no effect on i) *MSY1* or ii) *RSM7* transcript levels. All transcript levels were standardized to nuclear *ACT1* and expressed as percentage of wild-type (set at 100%) +/− standard error of the mean (n = 2).

The number of significant (Methods S1) Mito-nDNA interactions increased >10-fold in respiring (*i.e.* glycerol lactate grown) cells, relative to glucose or galactose grown cells (Table 1). This increase was not due to a higher number of sequence reads for the respiring sample. Thus, a greater number of unique nuclear loci connect to mtDNA during respiratory growth when the mitochondria are most active. This result, coupled with the need for a functional electron transport chain and reverse transcriptase machinery, led us to hypothesize that the Mito-nDNA interactions are functional in nature, and specifically that they are capable of controlling the transcript levels of the nuclear loci with which they interact. To test this we performed quantitative reverse transcriptase PCR (qRT-PCR) to determine the transcript levels of the nuclear encoded *MSY1* and *RSM7* genes in WT cells, the mitochondrial group-II intron knockout mutant (161-U7 GII-0), and strain 161-U7 GII-0 aI5γ (Figure 4A). We found that the population transcript level of the *MSY1* gene is significantly higher (t-test, two-sample unequal variance, one-tail, n = 2, p = 0.0007) in strain 161-U7 GII-0 (Figure 5A), which does not contain the probe site and, therefore, has no detectable *COX1*-*MSY1* interaction (Figure 4A and B), thus identifying the maximum transcript level in the absence of detectable inter-organelle interactions. Critically, we observed a similar population level increase in *MSY1* transcript levels following the removal of the type II introns, except aI5γ (*i.e.* strain 161-U7 GII-0 aI5γ; Figure 5A). A similar increase was observed for *RSM7* transcripts in both the 161-U7 GII-0 and 161-U7 GII-0 aI5γ strains relative to the WT (Figure 5B), consistent with the effects of intron deletion on the *Q0182*-*RMS7* interaction level (Figure 4C). By contrast deletion of *MRS1*, which is involved in mitochondrial group I intron splicing [36], [37], had no effect on either *MSY1* or *RSM7* transcript levels (Figure 5C), or the *COX1*-*MSY1* interaction frequency (Figure S5). Thus, strains lacking mitochondrial reverse transcriptase activity have lower frequencies of Mito-nDNA interactions and increased levels of nuclear encoded transcripts. These results suggest that cDNA mediated Mito-nDNA interactions are involved in the regulation of the nuclear transcripts, and therefore that the Mito-nDNA interactions we observed are biologically relevant.

The finding that inter-organelle interactions affect nuclear transcript levels necessarily predicts that the deletion of *yme1*, which reduces the frequency of the *COX1*-*MSY1* interaction (Figure 2), should correlate with an increase in nuclear *MSY1* transcript levels. Indeed increases in *MSY1* transcript levels, and other genes involved in mitochondrial gene expression and the biogenesis of the respiratory chain, have been identified within yeast cells containing the *yme1* deletion growing with a mixed respiro-fermentative metabolism [38].

## Discussion

In this study we have shown that a large number of nuclear loci interact strongly and reproducibly with Mitochondrial DNA in *S. cerevisiae* and that the spectrum of these interactions is dependent upon the carbon source on which the yeast are grown. Interestingly, we find that Mito-nDNA interactions are significantly reduced when group II mitochondrial introns that contain reverse transcriptase machinery are deleted. This suggests that the mitochondrial DNA that is involved in these inter-organelle interactions is cDNA that has been reverse transcribed from mitochondrial RNAs. Critically, we demonstrate that suppression of inter-organelle DNA-DNA interactions correlates with elevated transcript levels for the interacting nuclear gene and a reproducible albeit small increase in growth rate (Figure S6), suggesting that these interactions are biologically relevant and play a role in regulating nuclear gene expression. This is further supported by previous observations that yeast nuclear transcription responds to the presence or absence of mitochondrial genome sequences [18], [19]. From these results we propose that the Mito-nDNA interactions act as part of an inter-organelle communication system to signal mitochondrial metabolic state and regulate gene expression. While this DNA based inter-organelle communication may seem surprising, there is a large body of evidence demonstrating the presence of mitochondrial DNA in the nucleus and supporting the on-going nature of this transfer [7]–[9], [39]–[45]. Thus, it is plausible that the process of mitochondrial DNA transfer has evolved into a functional signaling mechanism. In the case of the glucose and galactose dependent *COX1-MSY1* and *Q0182*-*RSM7* interactions we have shown a repressive role for Mito-nDNA interactions in the control of nuclear transcript levels. However, there is no reason to assume that all interactions are repressive.

The observation that mito-nDNA interactions correlated with chromosome length would be consistent with non-specific interactions. However, this relationship is also consistent with the hypothesis that the interactions are enriched with elements which are themselves evenly distributed across the genome. Yeast genes, ARS's, and nuclear encoded mitochondrial genes fulfill this criterion (Figure S7). Importantly, we observed that the nuclear fragments involved in the interactions are enriched for regions that overlap genes with mitochondrial functions in glucose (p = 0.08) and glycerol-lactate grown cells (p<10^−8^, Table S3), in agreement with earlier observations [17]. The condition specific significance of these enrichments can be interpreted as reflecting the regulatory roles of these interactions as part of a functional signaling mechanism.

Our global network analyses were obtained using proximity-based ligation methodologies (i.e. GCC and q3C) with a single restriction enzyme (*i.e. Msp*I). Duan *et al.* used different restriction enzymes (*i.e. Eco*RI and *Hind*III) to interrogate yeast genome structure [46]. A comparison of the interaction frequencies between Duan *et al.*'s *Eco*RI results and ours showed that the data sets share a large portion of total interactions (Table S4) despite the fact that the interaction frequencies do not correlate quantitiatively (Figure S8). Duan *et al.'s* investigation of yeast genome organization did not report any Mito-nDNA interactions [46]. However, the absence of these interactions was due to the author's omission of the mitochondrial genome from their analyses because there is more than one copy of the mitochondrial genome per cell. This form of repetition is surmountable because interactions can be mapped onto a single scaffold, unlike elements which are repeated at different locations within the genome sequence. Re-examination of the datasets published by Duan *et al.* [46] identified a number of conserved Mito-nDNA between our datasets (Table S4). Interactions between *COX1*-*MSY1* and *Q0182*-*RSM7* (Data S1 and S2, respectively) were observed in both datasets, at levels above or just below the cut-off. The observation that Mito-nDNA interactions are present in these datasets is important for several reasons: 1) the methodologies while both based on proximity-ligation were considerably different, particularly with respect to enrichment and cell conditions; and 2) *Eco*RI cuts the yeast genome less frequently and at different positions to *Msp*I. While this goes some way towards relieving the likelihood that the interaction network is dependent on the restriction profile it does not completely alleviate this possibility, particularly because only one Mito-nDNA interaction was observed in the *Hin*dIII datasets (Data S3). The failure to identify more of these interactions in the *Hin*dIII datasets supports our conclusion that only parts of the mitochondrial genome are transferred into the nucleus and interact with the nuclear DNA. Because *Hin*dIII only cuts the yeast mitochondrial genome two times it is not unexpected that it does not cut within the transferred parts. Furthermore, the *Hin*dIII results support the finding that the interactions are not due to random ligation because random interactions would occur irrespective of fragment length.

It has been shown that DNA sequences which share stretches of sequence homology can associate *in vitro* [47], [48]. Theoretically it is possible that such a mechanism could allow the sequence dependent association of fragments prior to ligation in our experiments. However, only three pairs of interaction fragments that we detected showed any homology upon blast analysis (Data S6) and these were not the interactions that we investigated. Therefore, this mechanism is unlikely to contribute significantly to our results.

Deletion of the group II introns suggests a role for mitochondrial encoded RNA intermediates in the inter-organelle interactions. This implies that the regions of the mitochondria that are involved in these interactions should predominantly be open reading frames (ORFs). However, there was no bias for mitochondrial ORFs being involved in the Mito-nDNA or Mito-rDNA interactions we observed. Superficially, this argues against the transfer occurring through reverse-transcribed cDNAs. However, yeast mitochondrial genes are transcribed as polycistronic transcripts from 14 ATATAAGTA consensus promoters and possibly another 5 non-consensus promoter sites around the mitochondrial genome [49]. Hence, a large percentage of the mitochondrial genome is physically transcribed [50]–[52] and therefore available to act as a template for reverse transcription.

The incomplete ablation of the interactions following the deletion of the mitochondrial group II introns raises the possibility that not all Mito-nDNA interactions involve reverse-transcribed mitochondrial sequences. If mitochondrial mini-circles contribute to inter-organelle interactions [8], we should have seen an increase in interaction frequency in the Δ*yme1* mutant. The fact that we did not indicates that this form of mitochondrial DNA transfer has a negligible role in the signaling pathway we are proposing. Ongoing transfer despite the loss of the mitochondrial reverse transcriptase can be explained by: 1) cytoplasmic or nuclear reverse transcription of mRNA released from damaged mitochondria, or 2) the presence of other retrotransposon or retroviral encoded reverse-transcriptase of either mitochondrial or nuclear origin within the mitochondrial matrix. Such a mechanism is supported by the identification of remnants of nuclear derived *copia*-, *gypsy*- and LINE-like retrotransposon elements within *Arabidopsis* mitochondria [53], [54].

It is unclear whether the mtDNA that participates in the Mito-nDNA interactions is transferred by a direct connection between the mitochondrial and nuclear organelles or by uptake from the cytoplasm. Uptake from the cytoplasm is feasible given that unstable mitochondrial plasmids are first released into the cytoplasm [7], [9], and high success rates are generally attained for yeast transformation which involves passage through the cytoplasm [55], [56]. However, Ricchetti *et al.* demonstrated that the mtDNA mediated repair of nuclear double strand breaks is independent of Δ*yme1* mutations [6] and therefore occurs through another, possibly direct, transport mechanism. Direct transport from the mitochondria to nuclear compartments could occur as a result of a tethering/transport complex that physically links mitochondria to the endoplasmic reticulum [57].

In conclusion, several important questions are raised by this work. Firstly, what is the mechanism by which the Mito-nDNA interactions affect changes in transcript levels? Is the effect mediated by physical interaction between the mitochondrial derived cDNA and the nuclear locus or by more indirect means? It is possible that transcription factors may be sequestered or recruited to locations of activity through interactions with the mitochondrial DNA. Secondly, does this inter-organelle signaling pathway operate on a general level or function just to regulate conditionally essential genes (e.g. *RMS7* and *MSY1*)? Thirdly, while we demonstrated that specific interactions are dependent upon reverse transcription, whether this is true for all the Mito-nDNA and Mito-rDNA interactions remains to be determined. Finally, the universal significance of these interactions remains to be determined, particularly given the non-ubiquitous distribution of group II introns within higher eukaryotic mitochondria. The pervasive presence of reverse transcription within higher eukaryotic cells leads us to propose that this phenomenon will be widespread and that it deserves further investigation.

## Materials and Methods

### Strains and growth conditions

*Saccharomyces cerevisiae* strains (Table S5) were stored (−80°C) and cultured (30°C, 160 rpm) on synthetic complete (SC) medium containing amino acid supplements and glucose (2% w/v) [58], glycerol lactate (2% glycerol v/v 2% lactic acid v/v with 0.05% glucose w/v), or galactose (2% w/v). For Genome Conformation Capture (GCC) and Chromosome Conformation Capture (3 C) analyses, strains were recovered from −80°C on SC glucose (2% w/v) agar (2%) plates for 48 hours prior to starter culture inoculation. Starter cultures were grown (30°C, 160 rpm, 16 h) in SC glycerol lactate or glucose medium containing amino acid supplements, as indicated. Test cultures were inoculated, from the starter cultures into SC media (containing the indicated carbon source), grown (30°C, 160 rpm) and harvested at an optical density (OD_600_) of 0.6. Mitochondrial uncoupling was achieved by the addition of 2,4-Dinitrophenol (5 mM final concentration) for 45, 90, or 180 minutes (Figure S4). Cell cycle arrest was achieved by treatment (180 mins, 30°C, 160 rpm) with cell cycle inhibitors (*i.e.* α-factor (3.4 µm), nocodazole (15 µgml^−1^), or hydroxyurea (100 mM)). Cell cycle arrest was confirmed by microscopy.

### Genome Conformation Capture (GCC)

GCC was performed according to [17]. Refer to the supplementary methods for a detailed description. Briefly, chromatin was prepared from 15 sets of 10^8^ (*i.e.* a total of 1.36×10^9^) cross-linked cells. Chromatin was digested with *MspI* (Fermentas) and ligated (T4 ligase; Invitrogen). Crosslinks were reversed in the presence of proteinase K (final concentration 7–11 µg, Roche). Samples were treated with RNase A (final concentration 10 µgml^−1^) prior to purification by phenol∶chloroform (1∶1 v/v, three times) and column extraction (Zymo Clean and Concentrator, Zymo Research). Paired-end sequencing (36 bp) was performed on 5 µg DNA using the Illumina Genome Analyzer platform (Allan Wilson Centre, Massey University, New Zealand & Friedrich Miescher Institute for Biomedical Research, Basel, Switzerland).

External controls were added at two steps in the GCC protocol to control for random ligation events. The first ligation control, a linear DNA fragment with a free *MspI* site at one end (Methods S1), was added in a 1∶1 ratio with the nuclear genome prior to the addition of ligase. The second ligation control (1×10^6^ molecules of pUC19) was included prior to RNase A treatment as a control for the sequencing step ligation.

### GCC Network Assembly

Network assembly was performed using Topography v1.19 (available on request [17]). The SOAP [59] algorithm was used to position paired end sequences and single ends, which contain an *MspI* restriction site, onto the *S. cerevisiae* reference genome (Methods S1). No mismatches were allowed.

### Bioinformatic analyses

Bioinformatic and statistical analyses (see Methods S1) were performed on chromosomal interactions involving the nuclear and mitochondrial genomes for which the sequences mapped uniquely onto the reference genome. Connections with the ribosomal DNA (rDNA), 2 micron plasmid and mitochondrial genomes were considered as unique because they could be positioned to a ∼1 MB region of Chromosome XII, the 6318 bp 2 micron plasmid or the ∼85 kbp mitochondrial genome, respectively [17]. All statistical analyses involving 2 micron plasmid, mitochondrial, or rDNA sequences included copy number corrections (Methods S1). Other repetitive elements, such as LTRs and tRNAs, were omitted from the analysis.

### Chromosome Conformation Capture (3 C)

3 C samples were prepared as previously described [60]. Refer to Methods S1 for a detailed description. Quantitative 3 C analyses [17] were performed using FAM labeled BHQ Probes (BioSearch Technologies; Table S2) and Taqman® Gene Expression Master Mix (Applied Biosystems) on an ABI Prism 7000 Sequence Detection System (SDS7000). Chromosomal coordinates for the interactions under investigation are listed in Methods S1. Samples (2 µl in triplicate) were analyzed in a final reaction volume of 20 µl using primers listed in Table S2. Assays were performed using a 3-stage program (50°C, 2:00 min; 95°C, 10:00 min; 45×[95°C, 0:15 sec; 60°C, 1:00 min]).

Dedicated interaction standards (concentration from: 2 ng µl^−1^–2×10^−15^ g µl^−1^) were prepared by PCR amplification (from *S. cerevisiae* BY4741) of the interacting regions, followed by *MspI* digestion and ligation of the two interacting partner fragments. Mitochondrial and nuclear genome (*i.e. GAL1*) copy number were determined by qPCR (Table S2) using Sybr-green and a five stage program (50°C, 2:00 min; 95°C, 2:00 min; 40× [95°C, 0:15 sec; 59.5°C, 0:30 sec; 72°C, 0:30 sec]; 55°C, 1:00; followed by a dissociation analysis) on an ABI Prism 7000 Sequence Detection System (SDS7000). An *S. cerevisiae* BY4741 genomic DNA sample (concentration from: 2 ng µl^−1^–7.78125×10^−4^ ng µl^−1^) was used as a control for all Sybr-green assays.

For comparison, all samples were presented as a percentage of wild-type, following standardization for: 1) the amount of a15γ intron-containing DNA (*i.e.* mitochondrial copy number); or 2) the number of nuclear genomes (determined using the single copy *GAL1* locus; [61]; Primer sequences are listed in Table S2. This standardization was performed to correct for alterations to mitochondrial genome stability and the rates of appearance of rho^−^ or rho^0^ strains. This is critical as inter-organelle interactions are dependent upon the presence of the mitochondrial genome (see 161-U7 GII-0 results). The method of standardization depends upon the interaction being investigated (*i.e. COX1*-*MSY1* interactions were standardized by mitochondrial genome copy number while nuclear-nuclear locus interactions were standardized by *GAL1* copy number). No significant differences were observed when inter-organelle interactions were standardized by mitochondrial or nuclear copy number (data not shown).

### RNA extraction

Total RNA was extracted from *S. cerevisiae* grown in SC (Glucose) to an OD_600_ of 0.600. Briefly, cells were harvested (4,000 rpm, 4°C, 2 min) and washed with AE buffer (4,000 rpm, 4°C, 2 min; 50 mM Sodium Acetate, 10 mM EDTA, pH 5.3). The cell pellet was suspended in phenol/chloroform/isoamyl alcohol (400 µl, 24/24/1) and glass beads (400 µl). Cells were lysed in a bead mill (SPEX sample prep 2010, Geno/Grinder; 1,750 rpm, 8×30 sec cycles with 60 sec resting intervals at 4°C). Lysed cells were frozen (−80°C, 15 min), thawed and pelleted (15,000 rpm, 5 min, 4°C). The aqueous phase was extracted twice with phenol/chloroform/isoamyl alcohol (400 µl, 24/24/1). Total RNA was pelleted (15,000 rpm, 10 min, 4°C), following addition of 2/3 s volume of 8 M LiCl and freezing (−20°C, 2 h). RNA was washed (70% ethanol), and the pellet air-dried. Total RNA was suspended (60°C, 10 min) in 80 µl of DECP treated water (Invitrogen). DNA was removed from the total RNA samples (5 µg, 20 µl) by treatment with 1 µl of TURBO DNase (TURBO DNA-*free*™ Kit, Ambion) as per manufacturer's instructions. Samples were centrifuged (10,000 g, 1.5 min) and the supernatant was retained. Total RNA concentration was measured using a Nano-drop and 50 µl samples (50 ng/µl) were stored at −80°C.

### Quantitative Reverse Transcription-PCR

qRT-PCR standards were amplified from *S. cerevisiae* BY4741 genomic DNA (Table S2). PCR products were purified (Zymo DNA clean and concentrator™-5 kit according to manufacturer's instructions). The concentration of each qRT-PCR standard was determined by Nano-drop and used to make dilutions ranging from 4.0–4.0×10^−5^ ng/µl. qRT-PCR reactions were performed using One Step SYBR® Ex Taq™ qRT-PCR Kit according to the manufacturer's instructions (TaKaRa). The qRT-PCR was run with the following protocol: 42°C, 5 min; 95°C, 10 sec; 40× [95°C, 5 sec; 60°C, 31 sec] 95°C, 15 sec; 60°C, 1 min; 95°C, 15 sec. All transcript levels were standardized to nuclear *ACT1* and expressed as percentage of wild-type (set at 100%) +/− standard error of the mean.

### Supplementary files

The following additional data are available with the online version of this paper. Supplementary material file contains: Figures S1, S2, S3, S4, S5, S6, S7, S8, Tables S1, S2, S3, S4, S5, Methods S1 and Supplementary references. Additional data files: contain the analysis of the Duan data (Data S1, S2, S3, S4, S5); and the mitochondrial blast analyses (Data S6); a list of all uniquely mapping mito-nDNA and mito-mito interactions and their interaction strength (Data S7); a list of all mapped mito-nDNA and mito-mito interactions and their interaction strength (Data S8); and a list of nuclear genes with a mitochondrion annotation in gene ontology (Data S9). Sequences are available from GEO (accession number GSE34132).

## Supporting Information

Figure S1 **Biological Repeats correlate well at the** ***MspI*** **restriction fragment level.** Two biological repeats were performed for each condition; A) glucose, B) glycerol lactate, and C) galactose. R^2^ values are as follows; Glucose 0.78, glycerol lactate 0.93, and galactose 0.93. Scatter plots were constructed from statistically significant (p = <0.0004) interactions involving only *MspI* fragments which could be uniquely positioned on the reference genome. Adjacent interactions have been omitted as we are unable to distinguish between true adjacent interactions and those which are the result of simply sequencing across an uncut *MspI* site. Circularized fragments (*i.e.* self interactions) have also been omitted.
(DOC)

Figure S2 **Inter-organelle interactions vary with metabolic state and do not occur evenly across the mitochondrial genome.** Interaction frequency was graphed as a percentage of the total number of interactions in the sample, according to segment length. To test whether Mito-nDNA interactions have a uniform distribution (*i.e*, the total number of interactions in a segment is proportional to its length) we aggregated consecutive restriction fragments to create 58 sections that were expected to have at least 5 interactions under the null hypothesis of uniformity. A Chi-squared goodness of fit test was performed, and the distribution of the interactions was shown to deviate significantly from uniformity (p<0.0001, 57 df) for all conditions, Thus, Mito-nDNA interactions are not uniformly distributed across the mitochondrial genome. The linearized mitochondrial genome is shown for comparison of the interaction frequency with mitochondrial ORF and inter-genic sequence positions. Metabolic conditions were as follows: A) respiro-fermentation (glucose), B) respiro-fermentation (galactose), and C) respiration (glycerol lactate). Only statistically significant unique interactions between the mitochondrial genome and nuclear chromosomes were included in this analysis (p≤10^−5^; n = 2). Interactions with the rDNA and 2-micron plasmid were removed. D) Nuclear genome interactions are not enriched over mitochondrial open reading frames. We compared the numbers of nuclear genome interactions with mitochondrial inter- and intra-genic regions to determine if the interactions across the mitochondrial genome were enriched over the open reading frames (ORFs). Galactose displays a larger number of interactions with mitochondrial ORFs but the difference is not statistically significant. Interactions were assigned proportionally to inter- and intra-genic regions to obtain a ratio of inter-genic to intra-genic interactions and expressed as percentages. tRNAs were not deemed intra-genic. Interestingly, the galactose sample exhibited 7% and 13% more inter-organelle interactions involving the *COX1* ORF than glycerol lactate and glucose, respectively. Thus, while there is no obvious preference for interactions with mitochondrial ORFs, interactions involving *COX1* show differences between the datasets.
(DOC)

Figure S3 **The** ***COX1*** **-** ***MSY1*** **Mito-nDNA interaction is cell cycle dependent.** Cells were synchronized at three different cell cycle phases by treatment with α-factor (3.4 µm), Hydroxyurea (100 mM), or Nocodazole (15 µgml^−1^); G1, S, G2/M, respectively). Mito-nDNA interaction frequency, between the representative mitochondrial and nuclear *MspI* fragments, was assayed by quantitative 3 C (see Methods S1). Interaction values were corrected for mitochondrial genome copy number (see Methods). Interaction values are expressed as percentages of the untreated sample (set at 100%) +/− standard error of the mean (n = 3).
(DOC)

Figure S4 **5 mM 2,4-Dinitrophenol (DNP) inhibits respiratory growth but does not prevent growth of fermenting** ***S. cerevisiae*** **BY4741 cells.** *S. cerevisiae* BY4741 cultures were grown (50 ml, 30°C, 160 rpm) on glucose (fermentation) or glycerol/lactate (respiration) to an Optical density (600 nm; OD_600_) of 0.600. Cultures were diluted to an OD_600_ of 0.150 (50 ml final volume) in their respective media. 5 mM DNP (final concentration) was added to two of the cultures, while two remained untreated. The cell growth was monitored (OD_600_) for a further 11.5 hours, with the exception of the untreated glucose culture which was only grown for 4 hours.
(DOC)

Figure S5 **Deletion of** ***MRS1*** **(BY4741 Δ** ***mrs1*** **), a nuclear gene involved in splicing mitochondrial type-I introns, has no significant effect on the frequency of the** ***COX1-MSY1*** **interaction in glucose grown yeast cells.** Interaction frequency was expressed as percentages of the wild type *S. cerevisiae* strain BY4741 (WT, set at 100%) +/− standard error of the mean (n = 3).
(DOC)

Figure S6 **Deletion of group II introns results in an increase in growth rate.** Growth rates were determined for *Saccharomyces cerevisiae* strains (161-U7, 161-U7 GII0, and 161-U7 GII0 +aI5γ; Figure 4a) grown in SC+2% glucose (30°C and 160 rpm). Cultures were inoculated to an initial optical density (OD_600_) of 0.05 from overnight cultures. The OD_600_ was measured every two hours for 10 hour. Data represent the mean ± SD (n = 3).
(DOC)

Figure S7 **ARS and ORF numbers correlate with chromosome size.** Data on ARS and ORF numbers and chromosome size were taken from the Saccharomyces genome database Genome Inventory (as of Nov 03, 2011). The length of chromosome XII was calculated based on it containing only two copies of the rDNA repeat.
(DOC)

Figure S8 **Comparison of the total interaction frequencies for the Glucose derived GCC data (this study) and Duan** ***et al.*** *EcoRI* derived datasets.
(DOC)

Table S1 **Mitochondrial copy number calculations.**
(DOC)

Table S2 **Primers and probes used in this study.**
(DOC)

Table S3 **Nuclear fragments involved in mito-nDNA interactions are enriched for regions that overlap genes with mitochondrial functions.** The percentage of nuclear fragments that overlap with nuclear encoded mitochondrial genes within the complete genome was calculated and compared to the percentage of nuclear fragments involved in mito-nDNA interactions that overlap with nuclear encoded mitochondrial genes. A test of proportions (prop.test) was performed in R to determine whether the percentage difference is significant, p-values are shown.
(DOC)

Table S4 **Comparison between the** ***Eco*** **RI interaction set from Duan** ***et al.*** **** [**46**] **and the glucose set from this study.**
(DOC)

Table S5 **Strains used in this study.**
(DOC)

Methods S1 **This file contains supplementary information for methods used in this manuscript.**
(DOC)

Data S1 **This text file contains the significant interactions that were identified as occurring between the mitochondrial genome and the region surrounding the nuclear** ***MSY1*** **locus in the dataset prepared by Duan** ***et al.*** **** [**46**] **.** The number of instances for any interaction that had to be seen was set at > = 3 for the *Mse*1 and > = 4 for the *Msp*1 datasets.
(TXT)

Data S2 **This text file contains the significant interactions that were identified as occurring between the mitochondrial genome and the region surrounding the nuclear** ***RSM7*** **locus in the dataset prepared by Duan** ***et al.*** **** [**46**] **.** The number of instances for any interaction that had to be seen was set at > = 3 for the *Mse*1 and > = 4 for the *Msp*1 datasets.
(TXT)

Data S3 **This text file contains the one interaction that was identified as occurring between the mitochondrial and nuclear genomes in chromatin cut with** ***Hin*** **dIII and subsequently** ***Mse*** **1.**
(DAT)

Data S4 **This file contains all the interactions that were identified by re-analysis of the Duan** ***et al.*** **** [**46**] **datasets for chromatin digested with** ***Eco*** **RI and subsequently** ***Mse*** **1.** All listed interactions were all above the cut-off which was set at > = 3.
(DAT)

Data S5 **This file contains all the interactions that were identified by re-analysis of the Duan** ***et al.*** **** [**46**] **datasets for chromatin digested with** ***Eco*** **RI and subsequently** ***Msp*** **1.** All listed interactions were all above the cut-off which was set at > = 4.
(DAT)

Data S6 **This file contains the results of a blastN analysis of mito-nDNA interacting fragments.** Only three pairs of interaction fragments that we detected showed any homology upon blast analysis. The smaller restriction fragment from each interaction pair was compared to the longer fragment by blastn using default parameters. The length, score, and evalue for each comparison was recorded. Comparisons that showed no similarity were given an evalue score of 10.
(CSV)

Data S7 **This text file contains a list of all uniquely mapped mito-nDNA and mito-mito interactions and their interaction strength.**
(XLSX)

Data S8 **This text file contains a list of all mapped mito-nDNA and mito-mito interactions and their interaction strength.** The interactions that are included in this list include those which mapped uniquely and were repetitive.
(XLSX)

Data S9 **This text file contains a list of all genes that have a mitochondrion gene ontology annotation.** The gene list was obtained from YeastMine (http://yeastmine.yeastgenome.org/yeastmine/begin.do).
(CSV)
