# Supplementary material for: Mitochondrial-Nuclear DNA Interactions Contribute to the Regulation of Nuclear Transcript Levels as Part of the Inter-Organelle Communication System
Source: PLoS One. 2012 Jan 23;7(1):e30943. doi: 10.1371/journal.pone.0030943 (PMC3264656; doi:10.1371/journal.pone.0030943)
Supplement: Figure S1 — Biological Repeats correlate well at the MspI restriction fragment level. Two biological repeats were performed for each condition; A) glucose, B) glycerol lactate, and C) galactose. R2 values are as follows; Glucose 0.78, glycerol lactate 0.93, and galactose 0.93. Scatter plots were constructed from statistically significant (p = <0.0004) interactions involving only MspI fragments which could be uniquely positioned on the reference genome. Adjacent interactions have been omitted as we are unable to distinguish between true adjacent interactions and those which are the result of simply sequencing across an uncut MspI site. Circularized fragments (i.e. self interactions) have also been omitted. (DOC) [file pone.0030943.s001.doc]

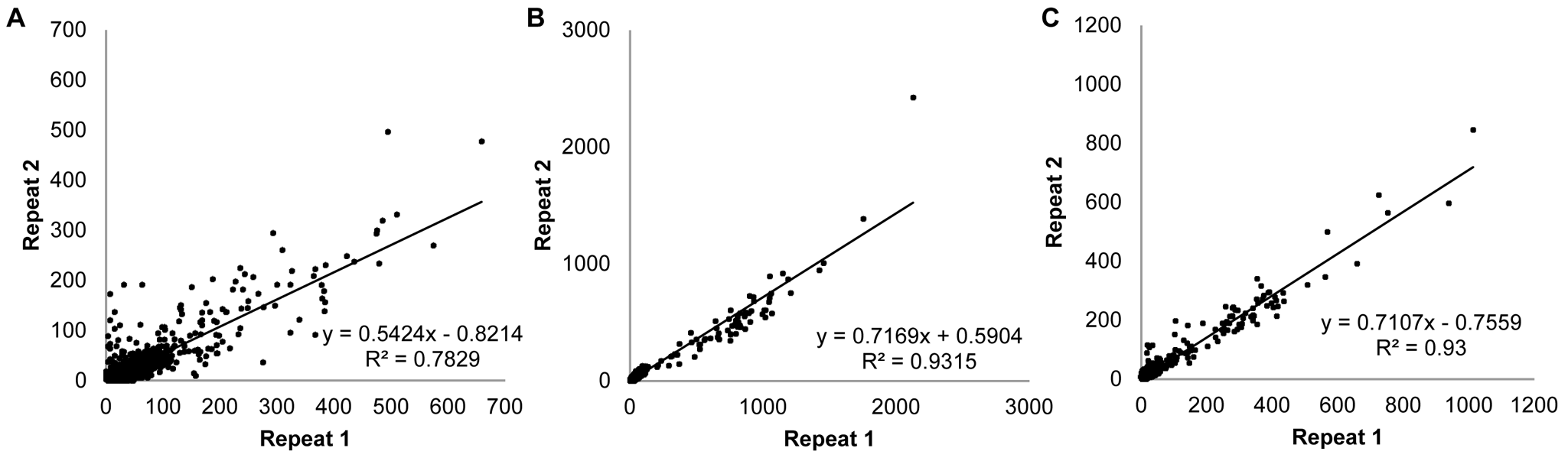
Figure S1: Biological Repeats correlate well at the *MspI* restriction fragment level. Two biological repeats were performed for each condition; A) glucose, B) glycerol lactate, and C) galactose. R^2^ values are as follows; Glucose 0.78, glycerol lactate 0.93, and galactose 0.93. Scatter plots were constructed from statistically significant (p=<0.0004) interactions involving only *MspI* fragments which could be uniquely positioned on the reference genome. Adjacent interactions have been omitted as we are unable to distinguish between true adjacent interactions and those which are the result of simply sequencing across an uncut *MspI* site. Circularized fragments (*i.e.* self interactions) have also been omitted.
