# Supplementary material for: Mitochondrial-Nuclear DNA Interactions Contribute to the Regulation of Nuclear Transcript Levels as Part of the Inter-Organelle Communication System
Source: PLoS One. 2012 Jan 23;7(1):e30943. doi: 10.1371/journal.pone.0030943 (PMC3264656; doi:10.1371/journal.pone.0030943)
Supplement: Figure S2 — Inter-organelle interactions vary with metabolic state and do not occur evenly across the mitochondrial genome. Interaction frequency was graphed as a percentage of the total number of interactions in the sample, according to segment length. To test whether Mito-nDNA interactions have a uniform distribution (i.e, the total number of interactions in a segment is proportional to its length) we aggregated consecutive restriction fragments to create 58 sections that were expected to have at least 5 interactions under the null hypothesis of uniformity. A Chi-squared goodness of fit test was performed, and the distribution of the interactions was shown to deviate significantly from uniformity (p<0.0001, 57 df) for all conditions, Thus, Mito-nDNA interactions are not uniformly distributed across the mitochondrial genome. The linearized mitochondrial genome is shown for comparison of the interaction frequency with mitochondrial ORF and inter-genic sequence positions. Metabolic conditions were as follows: A) respiro-fermentation (glucose), B) respiro-fermentation (galactose), and C) respiration (glycerol lactate). Only statistically significant unique interactions between the mitochondrial genome and nuclear chromosomes were included in this analysis (p≤10−5; n = 2). Interactions with the rDNA and 2-micron plasmid were removed. D) Nuclear genome interactions are not enriched over mitochondrial open reading frames. We compared the numbers of nuclear genome interactions with mitochondrial inter- and intra-genic regions to determine if the interactions across the mitochondrial genome were enriched over the open reading frames (ORFs). Galactose displays a larger number of interactions with mitochondrial ORFs but the difference is not statistically significant. Interactions were assigned proportionally to inter- and intra-genic regions to obtain a ratio of inter-genic to intra-genic interactions and expressed as percentages. tRNAs were not deemed intra-genic. Interestingly, the g [file pone.0030943.s002.doc]

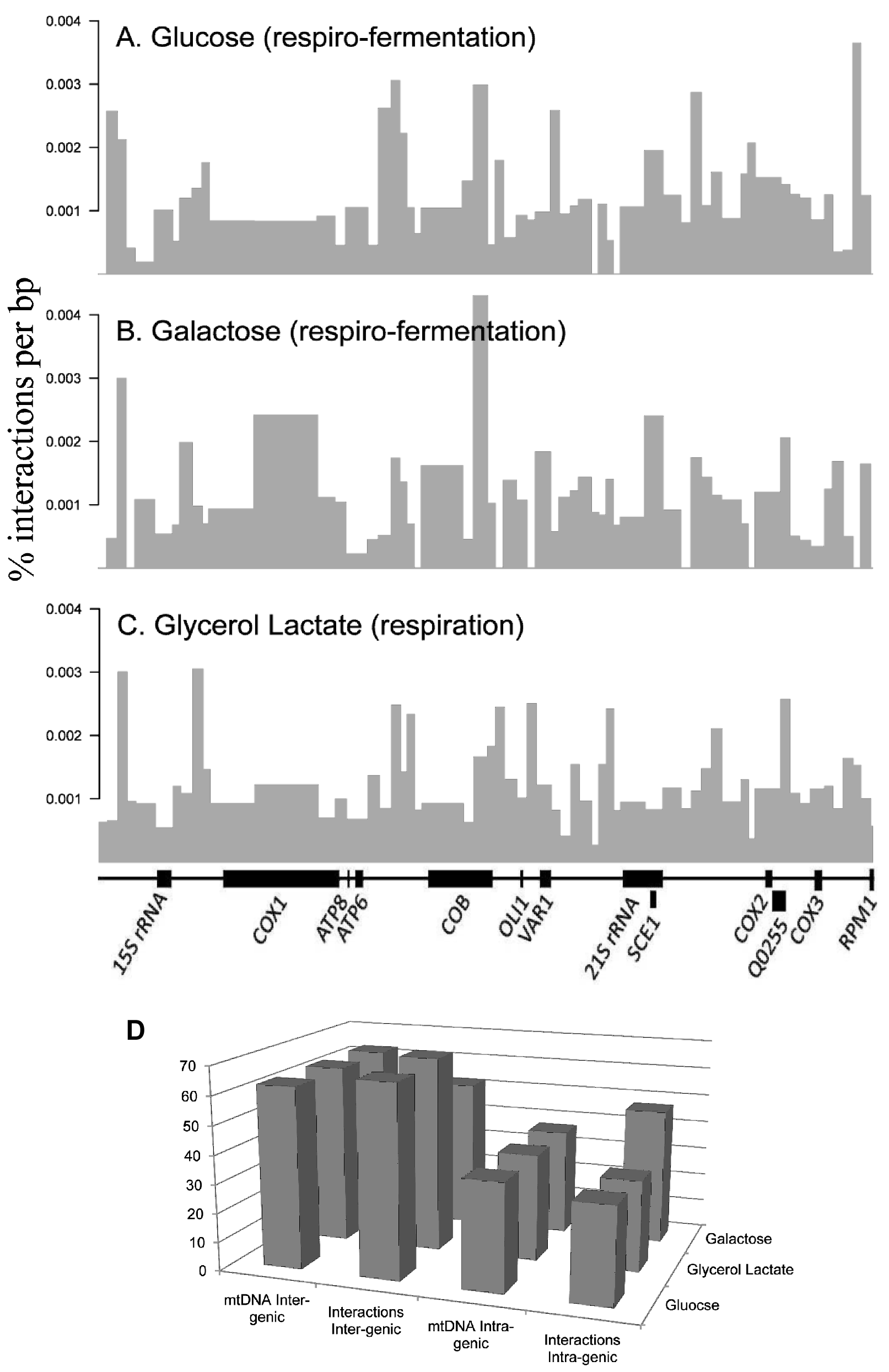


Figure S2: Inter-organelle interactions vary with metabolic state and do not occur evenly across the mitochondrial genome. Interaction frequency was graphed as a percentage of the total number of interactions in the sample, according to segment length. To test whether Mito-nDNA interactions have a uniform distribution (*i.e,* the total number of interactions in a segment is proportional to its length) we aggregated consecutive restriction fragments to create 58 sections that were expected to have at least 5 interactions under the null hypothesis of uniformity. A Chi-squared goodness of fit test was performed, and the distribution of the interactions was shown to deviate significantly from uniformity (p < 0.0001, 57 df) for all conditions, Thus, Mito-nDNA interactions are not uniformly distributed across the mitochondrial genome. The linearized mitochondrial genome is shown for comparison of the interaction frequency with mitochondrial ORF and inter-genic sequence positions. Metabolic conditions were as follows: A) respiro-fermentation (glucose), B) respiro-fermentation (galactose), and C) respiration (glycerol lactate). Only statistically significant unique interactions between the mitochondrial genome and nuclear chromosomes were included in this analysis (p≤10^-5^; n=2). Interactions with the rDNA and 2-micron plasmid were removed. D) Nuclear genome interactions are not enriched over mitochondrial open reading frames. We compared the numbers of nuclear genome interactions with mitochondrial inter- and intra-genic regions to determine if the interactions across the mitochondrial genome were enriched over the open reading frames (ORFs). Galactose displays a larger number of interactions with mitochondrial ORFs but the difference is not statistically significant. Interactions were assigned proportionally to inter- and intra-genic regions to obtain a ratio of inter-genic to intra-genic interactions and expressed as percentages. tRNAs were not deemed intra-genic. Interestingly, the galactose sample exhibited 7% and 13% more inter-organelle interactions involving the *COX1* ORF than glycerol lactate and glucose, respectively. Thus, while there is no obvious preference for interactions with mitochondrial ORFs, interactions involving *COX1* show differences between the datasets.
