# Supplementary material for: Mitochondrial-Nuclear DNA Interactions Contribute to the Regulation of Nuclear Transcript Levels as Part of the Inter-Organelle Communication System
Source: PLoS One. 2012 Jan 23;7(1):e30943. doi: 10.1371/journal.pone.0030943 (PMC3264656; doi:10.1371/journal.pone.0030943)
Supplement: Figure S3 — The COX1 - MSY1 Mito-nDNA interaction is cell cycle dependent. Cells were synchronized at three different cell cycle phases by treatment with α-factor (3.4 µm), Hydroxyurea (100 mM), or Nocodazole (15 µgml−1); G1, S, G2/M, respectively). Mito-nDNA interaction frequency, between the representative mitochondrial and nuclear MspI fragments, was assayed by quantitative 3 C (see Methods S1). Interaction values were corrected for mitochondrial genome copy number (see Methods). Interaction values are expressed as percentages of the untreated sample (set at 100%) +/− standard error of the mean (n = 3). (DOC) [file pone.0030943.s003.doc]

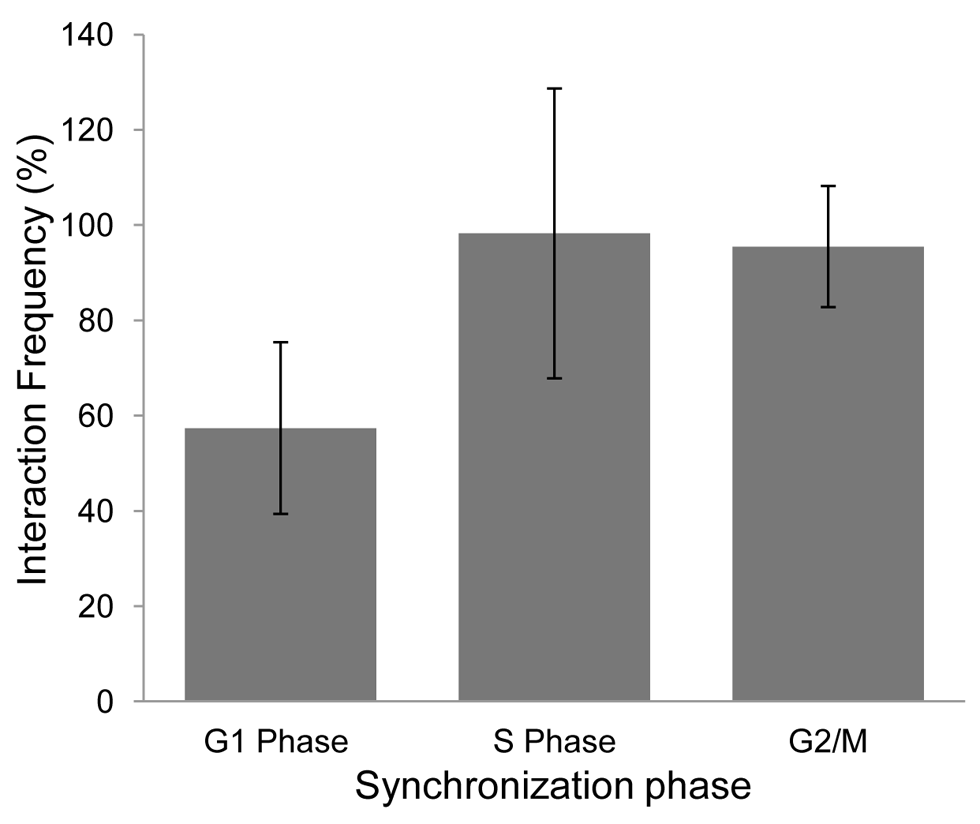
Figure S3: The *COX1*-*MSY1* Mito-nDNA interaction is cell cycle dependent. Cells were synchronized at three different cell cycle phases by treatment with α-factor (3.4µm), Hydroxyurea (100mM), or Nocodazole (15µgml^-1^); G1, S, G2/M, respectively). Mito-nDNA interaction frequency, between the representative mitochondrial and nuclear *MspI* fragments, was assayed by quantitative 3C (see Supplementary Methods). Interaction values were corrected for mitochondrial genome copy number (see Methods). Interaction values are expressed as percentages of the untreated sample (set at 100%) +/- standard error of the mean (n=3).
