# Supplementary material for: Mitochondrial-Nuclear DNA Interactions Contribute to the Regulation of Nuclear Transcript Levels as Part of the Inter-Organelle Communication System
Source: PLoS One. 2012 Jan 23;7(1):e30943. doi: 10.1371/journal.pone.0030943 (PMC3264656; doi:10.1371/journal.pone.0030943)
Supplement: Figure S4 — 5 mM 2,4-Dinitrophenol (DNP) inhibits respiratory growth but does not prevent growth of fermenting S. cerevisiae BY4741 cells. S. cerevisiae BY4741 cultures were grown (50 ml, 30°C, 160 rpm) on glucose (fermentation) or glycerol/lactate (respiration) to an Optical density (600 nm; OD600) of 0.600. Cultures were diluted to an OD600 of 0.150 (50 ml final volume) in their respective media. 5 mM DNP (final concentration) was added to two of the cultures, while two remained untreated. The cell growth was monitored (OD600) for a further 11.5 hours, with the exception of the untreated glucose culture which was only grown for 4 hours. (DOC) [file pone.0030943.s004.doc]

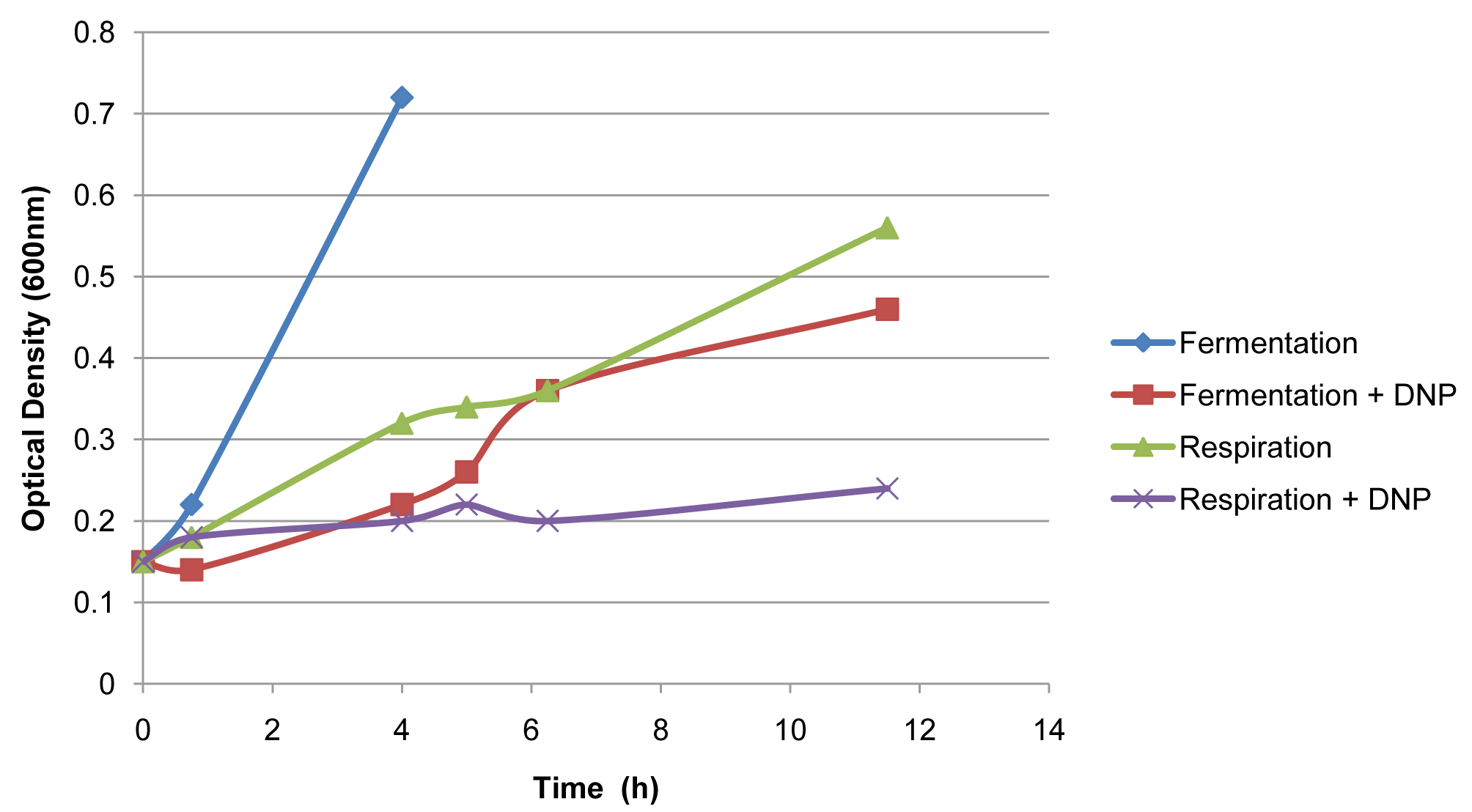


Figure S4: 5mM 2,4-Dinitrophenol (DNP) inhibits respiratory growth but does not prevent growth of fermenting *S. cerevisiae* BY4741 cells. *S. cerevisiae* BY4741 cultures were grown (50 ml, 30°C, 160 rpm) on glucose (fermentation) or glycerol/lactate (respiration) to an Optical density (600nm; OD_600_) of 0.600. Cultures were diluted to an OD_600_ of 0.150 (50 ml final volume) in their respective media. 5mM DNP (final concentration) was added to two of the cultures, while two remained untreated. The cell growth was monitored (OD_600_) for a further 11.5 hours, with the exception of the untreated glucose culture which was only grown for 4 hours.
