# Supplementary material for: Mitochondrial-Nuclear DNA Interactions Contribute to the Regulation of Nuclear Transcript Levels as Part of the Inter-Organelle Communication System
Source: PLoS One. 2012 Jan 23;7(1):e30943. doi: 10.1371/journal.pone.0030943 (PMC3264656; doi:10.1371/journal.pone.0030943)
Supplement: Figure S5 — Deletion of MRS1 (BY4741 Δ mrs1 ), a nuclear gene involved in splicing mitochondrial type-I introns, has no significant effect on the frequency of the COX1-MSY1 interaction in glucose grown yeast cells. Interaction frequency was expressed as percentages of the wild type S. cerevisiae strain BY4741 (WT, set at 100%) +/− standard error of the mean (n = 3). (DOC) [file pone.0030943.s005.doc]

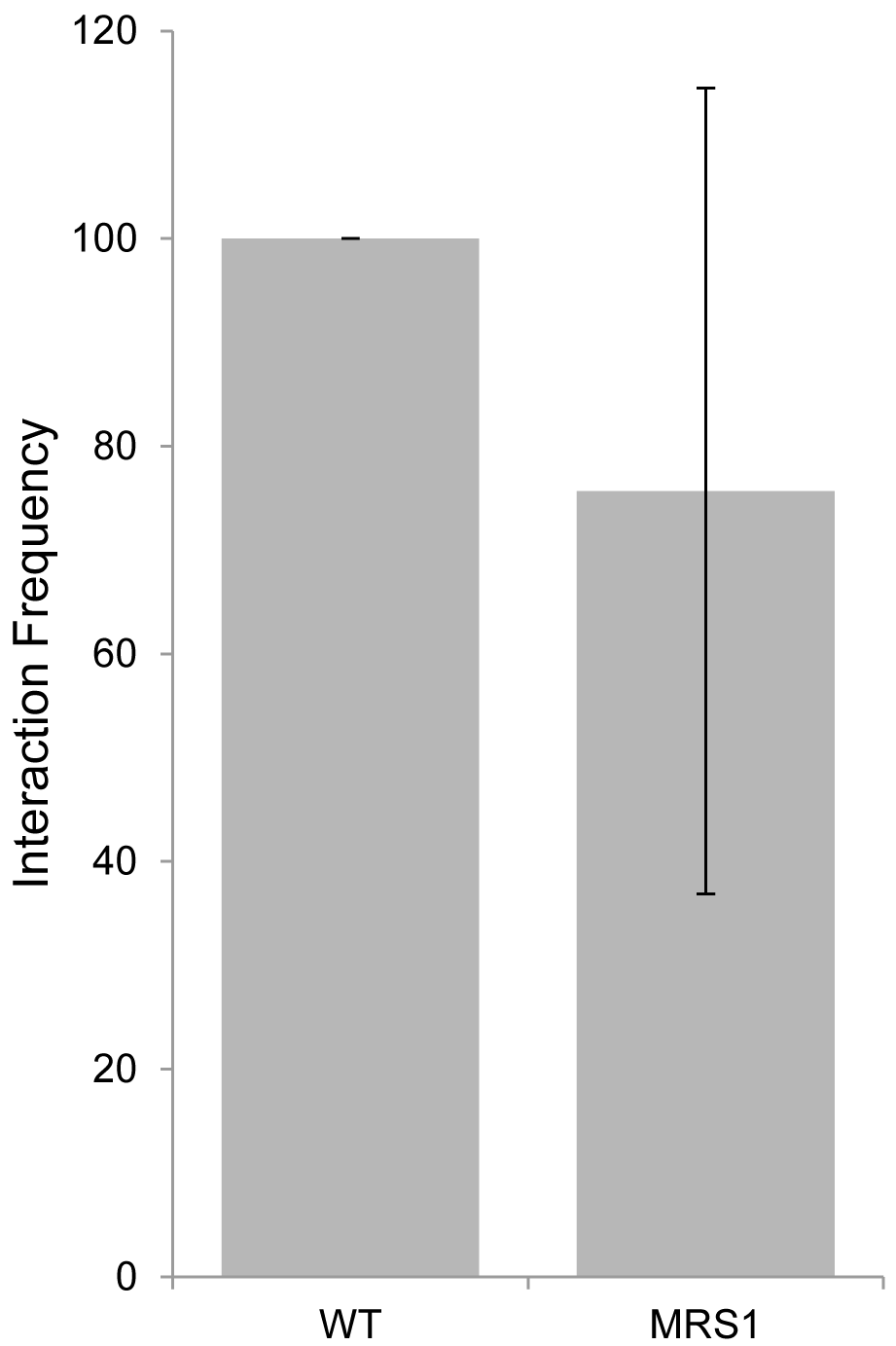


Figure S5: Deletion of *MRS1* (BY4741 *Δmrs1*)*,* a nuclear gene involved in splicing mitochondrial type-I introns, has no significant effect on the frequency of the *COX1-MSY1* interaction in glucose grown yeast cells. Interaction frequency was expressed as percentages of the wild type *S. cerevisiae* strain BY4741 (WT, set at 100%) +/- standard error of the mean (n=3).
