# Supplementary material for: Mitochondrial-Nuclear DNA Interactions Contribute to the Regulation of Nuclear Transcript Levels as Part of the Inter-Organelle Communication System
Source: PLoS One. 2012 Jan 23;7(1):e30943. doi: 10.1371/journal.pone.0030943 (PMC3264656; doi:10.1371/journal.pone.0030943)
Supplement: Figure S6 — Deletion of group II introns results in an increase in growth rate. Growth rates were determined for Saccharomyces cerevisiae strains (161-U7, 161-U7 GII0, and 161-U7 GII0 +aI5γ; Figure 4a) grown in SC+2% glucose (30°C and 160 rpm). Cultures were inoculated to an initial optical density (OD600) of 0.05 from overnight cultures. The OD600 was measured every two hours for 10 hour. Data represent the mean ± SD (n = 3). (DOC) [file pone.0030943.s006.doc]

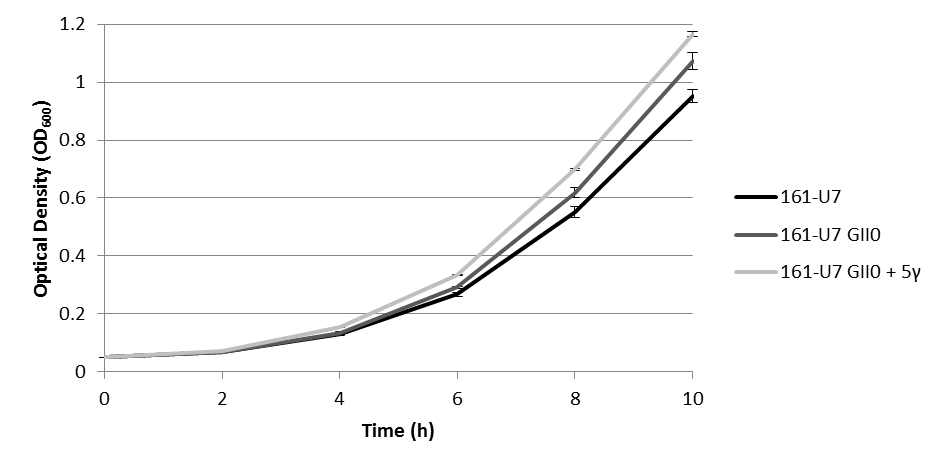


Figure S6: Deletion of group II introns results in an increase in growth rate. Growth rates were determined for *Saccharomyces cerevisiae* strains (161-U7, 161-U7 GII0, and 161-U7 GII0 +aI5γ; Figure 4a) grown in SC + 2% glucose (30°C and 160rpm). Cultures were inoculated to an initial optical density (OD_600_) of 0.05 from overnight cultures. The OD_600_ was measured every two hours for 10 hour. Data represent the mean ± SD (n=3).
