# Supplementary material for: Mitochondrial-Nuclear DNA Interactions Contribute to the Regulation of Nuclear Transcript Levels as Part of the Inter-Organelle Communication System
Source: PLoS One. 2012 Jan 23;7(1):e30943. doi: 10.1371/journal.pone.0030943 (PMC3264656; doi:10.1371/journal.pone.0030943)
Supplement: Figure S7 — ARS and ORF numbers correlate with chromosome size. Data on ARS and ORF numbers and chromosome size were taken from the Saccharomyces genome database Genome Inventory (as of Nov 03, 2011). The length of chromosome XII was calculated based on it containing only two copies of the rDNA repeat. (DOC) [file pone.0030943.s007.doc]

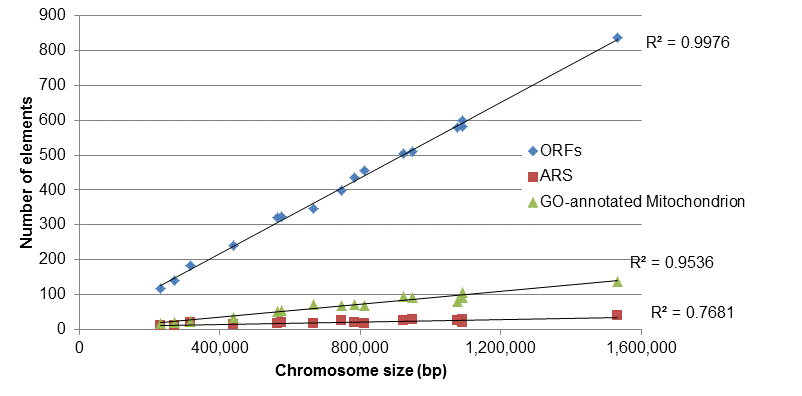
Figure S7: Numbers of ARS elements, ORFs, and genes with a mitochondrion gene ontology label correlate with chromosome size. Data on ARS and ORF numbers and chromosome size were taken from the Saccharomyces genome database Genome Inventory (Nov 03, 2011) and include all ARS and gene sequences (confirmed and putative). Genes with a mitochondrion gene ontology label were extracted using Yeastmine (<http://yeastmine.yeastgenome.org/yeastmine/template.do?name=GOTerm_Genes>; Table S4). The length of chromosome XII was calculated based on it containing only two copies of the rDNA repeat.
