# Supplementary material for: Mitochondrial-Nuclear DNA Interactions Contribute to the Regulation of Nuclear Transcript Levels as Part of the Inter-Organelle Communication System
Source: PLoS One. 2012 Jan 23;7(1):e30943. doi: 10.1371/journal.pone.0030943 (PMC3264656; doi:10.1371/journal.pone.0030943)
Supplement: Figure S8 — Comparison of the total interaction frequencies for the Glucose derived GCC data (this study) and Duan et al. EcoRI derived datasets. (DOC) [file pone.0030943.s008.doc]

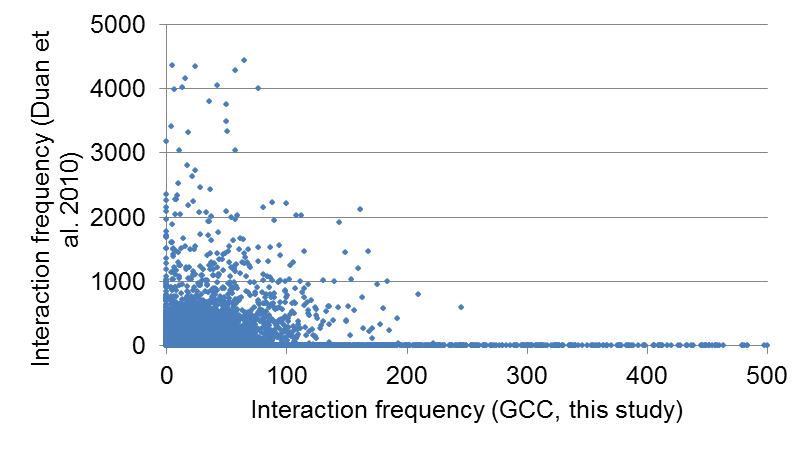


Figure S8: Comparison of the total interaction frequencies for the Glucose derived GCC data (this study) and Duan *et al.* *Eco*RI derived datasets. While there is no quantitative correlation between the interaction frequencies, the datasets shared a large number of interacting pairs (Table S4). Datasets were analysed as described (Supplementary Methods).

**References**

1. Duan Z, Andronescu M, Schutz K, McIlwain S, Kim YJ, et al. (2010) A three-dimensional model of the yeast genome. Nature 465: 363-367.
