# Supplementary material for: Mitochondrial-Nuclear DNA Interactions Contribute to the Regulation of Nuclear Transcript Levels as Part of the Inter-Organelle Communication System
Source: PLoS One. 2012 Jan 23;7(1):e30943. doi: 10.1371/journal.pone.0030943 (PMC3264656; doi:10.1371/journal.pone.0030943)
Supplement: Table S1 — Mitochondrial copy number calculations. (DOC) [file pone.0030943.s009.doc]

**Table S1:** Mitochondrial copy number calculations

| Condition | Total No.  sequence reads | No. *GAL1*  aligned reads | % total *GAL1* | No. AI5γ  aligned reads | % total  AI5γ | Ratio  AI5γ:GAL1 |
| --- | --- | --- | --- | --- | --- | --- |
| Glucose | 112335584 | 733 | 0.00065 | 8937 | 0.00796 | 12.19 |
| Glycerol Lactate | 98269812 | 767 | 0.00078 | 12156 | 0.01237 | 15.85 |
| Galactose | 96838770 | 621 | 0.00064 | 12808 | 0.01323 | 20.62 |
