# Supplementary material for: Mitochondrial-Nuclear DNA Interactions Contribute to the Regulation of Nuclear Transcript Levels as Part of the Inter-Organelle Communication System
Source: PLoS One. 2012 Jan 23;7(1):e30943. doi: 10.1371/journal.pone.0030943 (PMC3264656; doi:10.1371/journal.pone.0030943)
Supplement: Table S2 — Primers and probes used in this study. (DOC) [file pone.0030943.s010.doc]

**Table S2: Primers and probes used in this study.**

| **Name** | **Sequence** | **Comments** |
| --- | --- | --- |
| **MITOCHONDRIA COPY NUMBER** | | |
| **Mito+ve13909F** | **TGCTCAACGAAAGTGAATCAA** |  |
| **Mito(CNC)R13909** | GATTTATCGTATGCTCATTTCCAA |  |
| **NUCLEAR GENOME COPY NUMBER** | | |
| GAL1F | TTGCGAACACCCTTGTTGTA |  |
| GAL1R | CGTGCTCGATCCTTCTTTTC |  |
| **MITO-rDNA 3C TAQMAN** | | |
| MitogDNA3CForward | GTGAGCCGTATGCGATGAAAG |  |
| Nts1_599R | TTATTCCTTCCCGCTTTCCT |  |
| MitogDNA3CProbe | FAM-TCGCACGTACGGTTCTTACCGG |  |
| **MITO-rDNA POSITIVES** | | |
| rDNA+ve460025F | CATTATGCTCATTGGGTTGC |  |
| rDNA+ve460025R | AGGAAAGCGGGAAGGAATAA |  |
| **Mito+ve13909F** | **TGCTCAACGAAAGTGAATCAA** |  |
| MitoAcross13909R | TCCCGATAGGTAGACCTTTACAA |  |
| **MITOCHONDRIAL GROUP I AND II MUTANT GENOTYPE TESTING** | | |
| Mito_AI1_E&I_F | AGTGGTATGGCAGGAACAGC |  |
| Mito_AI1_E&I_R | CCCCGTAAAGTTAGCCCCTA |  |
| Mito_AI2_I_F | GGGGATTGTGATTCATGCTT |  |
| Mito_AI2_I_R | CTGTCTTCCTTCCTTGCATTT |  |
| Mito_AI3_I_F | ACTTTCTTCCCCTCCGAATC |  |
| Mito_AI3_I_R | GGCCCTCGTGGGGATAATA |  |
| Mito_AI4_I_F | TGATCAATTTTCATTACAGCGTTC |  |
| Mito_AI4_I_R | TTTTCTTGTAGTCTCTGAGGATCTTTT |  |
| Mito_AI5_I_F | AGGCAAACTCGAGGAAAACC |  |
| Mito_AI5_I_R | AATATCCTCAATTAAGAGGTCGAA |  |
| **nDNA-nDNA 3C TAQMAN** | | |
| gDNAgDNA3CForward | TGACACCGTCTCTTGTTTAGGA |  |
| gDNAgDNA3CReverse | TTGATCGTATCCTTCTCTAGTGAAC |  |
| gDNAgDNA3CProbe | FAM-TTAACTTCAGTTAAATCTTCAA |  |
| **Mito-nDNA 3C TAQMAN** | | |
| MitogDNA3CForward | GTGAGCCGTATGCGATGAAAG |  |
| MitogDNA3CR13221 | GAATCCCTCGCCAACATAGA |  |
| MitogDNA3CProbe | FAM-TCGCACGTACGGTTCTTACCGG |  |
| **LIGATION CONTROLS** | | |
| E.coli211bp3’MspIF | GCCAGAAATTCGTCGGTAAG |  |
| E.coli211bp3’MspIR | AACCGGTCATTGAAGTATTGA |  |
| Lambda185bp3’MspIF | TTTACAGCGTGATGGAGCAG |  |
| Lambda185bp3’MspIR | ACCAATCCAGCCGGTCAG |  |
| **Mito-nDNA POSITIVES** | | |
| **Mito+ve13909F** | **TGCTCAACGAAAGTGAATCAA** |  |
| MitoAcross13909R | TCCCGATAGGTAGACCTTTACAA |  |
| **MitogDNA3CR13221** | **GAATCCCTCGCCAACATAGA** |  |
| **gDNA+ve13221F** | CATCCATCTCGCAGCAATTA |  |
| **nDNA-nDNA POSITIVES** | | |
| gDNAAcross5313F | TGATCGTTGGGTAATGCTCTT |  |
| gDNAAcross5313R | AGCCTGTGGTTGATGGAAAC |  |
| gDNAAcross6434F | CCACCACAAATCAAGCCTCT |  |
| gDNAAcross6434R | TATGGTCGCTCCTGCCTATC |  |
| **RNA qRT-PCR** |  |  |
| RSM7_qRT-PCR_For | TGTCATTCCTGTGCCTCTGA |  |
| RSM7_qRT-PCR_Rev | TGGCTGTCTTGTGAATCTGG |  |
| MSY1_qRT-PCR_For | CGGCGTATGATGTTTACCAG |  |
| MSY1_qRT-PCR_Rev | CCGGAGCCAACTCCATATAA |  |
| ACT1_qRT-PCR_cont_F | ACATCGTTATGTCCGGTGGT |  |
| ACT1_qRT-PCR_cont_R | AGATGGACCACTTTCGTCGT |  |

Standard primers were designed using Primer3. Taqman primer and probes were designed using BioSearch Technologies RealTimeDesign online software.
