# Supplementary material for: Mitochondrial-Nuclear DNA Interactions Contribute to the Regulation of Nuclear Transcript Levels as Part of the Inter-Organelle Communication System
Source: PLoS One. 2012 Jan 23;7(1):e30943. doi: 10.1371/journal.pone.0030943 (PMC3264656; doi:10.1371/journal.pone.0030943)
Supplement: Table S3 — Nuclear fragments involved in mito-nDNA interactions are enriched for regions that overlap genes with mitochondrial functions. The percentage of nuclear fragments that overlap with nuclear encoded mitochondrial genes within the complete genome was calculated and compared to the percentage of nuclear fragments involved in mito-nDNA interactions that overlap with nuclear encoded mitochondrial genes. A test of proportions (prop.test) was performed in R to determine whether the percentage difference is significant, p-values are shown. (DOC) [file pone.0030943.s011.doc]

**Table S3 Nuclear fragments involved in mito-nDNA interactions are enriched for regions that overlap genes with mitochondrial functions.**

|  | Total number of fragments | Number of fragments that overlap with nuclear encoded mitochondrial genes | Percentage (%) |
| --- | --- | --- | --- |
| Complete genome | | | |
| Restriction enzyme fragments | 13847 | 3210 | 23.18 |
| Glucose condition | | | |
| Nuclear fragments involved in mito-nDNA interactions | 96 | 30 | 31.25  (p = 0.08116) |
| Galactose condition | | | |
| Nuclear fragments involved in mito-nDNA interactions | 77 | 23 | 29.87  (p = 0.211) |
| Glycerol-Lactate condition | | | |
| Nuclear fragments involved in mito-nDNA interactions | 1138 | 353 | 31.02  (p = 2.961e-09) |

The percentage of nuclear fragments that overlap with nuclear encoded mitochondrial genes (Data S9) within the complete genome was calculated and compared to the percentage of nuclear fragments involved in mito-nDNA interactions that overlap with nuclear encoded mitochondrial genes. A test of proportions (prop.test) was performed in R to determine whether the percentage difference is significant, p-values are shown.
