# Supplementary material for: Mitochondrial-Nuclear DNA Interactions Contribute to the Regulation of Nuclear Transcript Levels as Part of the Inter-Organelle Communication System
Source: PLoS One. 2012 Jan 23;7(1):e30943. doi: 10.1371/journal.pone.0030943 (PMC3264656; doi:10.1371/journal.pone.0030943)
Supplement: Table S4 — Comparison between the Eco RI interaction set from Duan et al. [46] and the glucose set from this study. (DOC) [file pone.0030943.s012.doc]

**Table S4: Comparison between the *Eco*RI interaction set from Duan *et al*. and the glucose set from this study.**

|  |  |  |  |  |  |  | Interactions | |
| --- | --- | --- | --- | --- | --- | --- | --- | --- |
|  |  |  |  |  |  |  | Total | mito-nDNA |
| Number of interacting pairs^a^ in glucose GCC set (this study): | | | | | | | 28254 | 1379 |
| Number of interacting pairs found by Duan *et al.*^b^: | | | | | | | 193351 | 1752 |
| Total number of interacting pairs^c^: | | | | | | | 202275 | 2889 |
|  | | | | | | |  |  |
| Number of shared interaction pairs between the data sets: | | | | | | | 19330 | 242 |
| Number of interaction pairs unique to the GCC data set: | | | | | | | 8924 | 1137 |
| Number of interaction pairs unique to the Duan *et al.* data set: | | | | | | | 174021 | 1510 |
|  |  |  |  |  |  |  |  |  |
| Fraction of GCC interaction pairs reproduced by Duan *et al*.: | | | | | | | 0.68 | 0.18 |
| Fraction of Duan *et al.* interaction pairs reproduced by GCC data set: | | | | | | | 0.10 | 0.14 |
| Fraction of Duan *et al.’*s 28254 strongest interaction pairs in the GCC data set: | | | | | | | 0.51 | - |
| Fraction of Duan *et al.'*s 1379 strongest mito-nDNA pairs in the GCC data set: | | | | | | | - | 0.15 |

^a^ Interacting *Msp*I fragment pairs were mapped onto an *Eco*RI fragmented genome (Supplementary Methods).

^b^ All *Eco*RI datasets were pooled for this analysis.

^c^ The union of the *Eco*RI mapped GCC and Duan *et al.* interaction sets.

Noise cut-offs for the total dataset were determined to be 3 (GCC) and 5 (Duan *et al.*), therefore interactions which were identified ≥3 times in the GCC, or ≥5 times in the Duan *et al*. dataset were used in this analysis. Noise cut-offs for the Mito-nDNA interactions were calculated as ≥5 and ≥6 for the GCC and Duan *et al*. datasets, respectively.

## References

1. Duan Z, Andronescu M, Schutz K, McIlwain S, Kim YJ, et al. (2010) A three-dimensional model of the yeast genome. Nature 465: 363-367.
