# Supplementary material for: Mitochondrial-Nuclear DNA Interactions Contribute to the Regulation of Nuclear Transcript Levels as Part of the Inter-Organelle Communication System
Source: PLoS One. 2012 Jan 23;7(1):e30943. doi: 10.1371/journal.pone.0030943 (PMC3264656; doi:10.1371/journal.pone.0030943)
Supplement: Table S5 — Strains used in this study. (DOC) [file pone.0030943.s013.doc]

**Table S5: Strains used in this study.**

| **Strain** | **Genotype and Comments** | **Background** |
| --- | --- | --- |
| **BY4741** | Mat**a** his3Δ1 leu2Δ0 met15Δ0 ura3Δ0 | Wildtype |
| **161-U7** | 1^+^2^+^ [MATa, ade1, lys1, ura3]. Wildtype | 161-U7 |
| **161-U7 GII-0 aI5γ** | GII-0 [MATa, ade1, lys1, ura3]. No group II introns aI5γ retained | 161-U7 |
| **161-U7 GII-0** | GII-0 [MATa, ade1, lys1, ura3]. No group II introns | 161-U7 |
| ***yme1*** | Mat**a** his3Δ1 leu2Δ0 met15Δ0 ura3Δ0 yme1∆::kanMX4 | BY4741 |
| ***yme2*** | Mat**a** his3Δ1 leu2Δ0 met15Δ0 ura3Δ0 yme2∆::kanMX4 | BY4741 |
| ***mdv1*** | Mat**a** his3Δ1 leu2Δ0 met15Δ0 ura3Δ0 mdv1∆::kanMX4 | BY4741 |
| ***mrs1*** | Mat**a** his3Δ1 leu2Δ0 met15Δ0 ura3Δ0 mrs1∆::kanMX4 | BY4741 |
| ***ade2*** | Mat**a** his3Δ1 leu2Δ0 met15Δ0 ura3Δ0 ade2∆::kanMX4 | BY4741 |

## References

1. Moran JV, Zimmerly S, Eskes R, Kennell JC, Lambowitz AM, et al. (1995) Mobile group II introns of yeast mitochondrial DNA are novel site-specific retroelements. Mol Cell Biol 15: 2828-2838.

2. Boulanger SC, Belcher SM, Schmidt U, Dib-Hajj SD, Schmidt T, et al. (1995) Studies of point mutants define three essential paired nucleotides in the domain 5 substructure of a group II intron. Molecular and Cellular Biology 15: 4479-4488.

3. Peebles CL, Belcher SM, Zhang M, Dietrich RC, Perlman PS (1993) Mutation of the conserved first nucleotide of a group II intron from yeast mitochondrial DNA reduces the rate but allows accurate splicing. Journal of Biological Chemistry 268: 11929-11938.
