# Supplementary material for: Mitochondrial-Nuclear DNA Interactions Contribute to the Regulation of Nuclear Transcript Levels as Part of the Inter-Organelle Communication System
Source: PLoS One. 2012 Jan 23;7(1):e30943. doi: 10.1371/journal.pone.0030943 (PMC3264656; doi:10.1371/journal.pone.0030943)
Supplement: Methods S1 — This file contains supplementary information for methods used in this manuscript. (DOC) [file pone.0030943.s014.doc]

# Supplementary methods

**Mitochondrial-nuclear DNA interactions regulate nuclear transcription.**

C.D.M. Rodley^a^, R.S. Grand^a^, B. Jones^b^, J.M O’Sullivan^aǂ^

^a^Institute of Natural Sciences, Massey University, Albany, New Zealand

^b^Centre for Mathematical Biology, Massey University, Albany, New Zealand

^ǂ^ Corresponding Author:

Justin M. O’Sullivan: Email [J.M.OSullivan@massey.ac.nz](mailto:J.M.OSullivan@massey.ac.nz); fax +64 9 441 8142

Address: Building 11

Station Crescent Gate 4, Oteha Rohe

Old Albany Highway

Albany, Auckland

New Zealand

## Methodology for Capturing Chromosomal Interactions

### *Harvesting cells and chromatin preparation*

*S. cerevisiae* cells were cultured (30°C, 160rpm) to an OD_600_ of 0.600. Cultures were cross-linked in formaldehyde (1% final v/v, 10 min, RT). Cells were pelleted (3000 rpm, 3 mins, 4°C) before being washed twice in wash buffer (5 ml, 3000 rpm, 3 mins, 4°C) and suspended in 400 µl FA lysis buffer (0.05 M Hepes KOH [pH 8.0], 0.14 M NaCl, 1 mM EDTA, 1% [v/v] Triton X-100, 0.1% DOC, mini complete EDTS-free protease inhibitors [11836170001 Roche]). Cells were counted and aliquoted into residues of 0.95x10^9^. Acid washed beads (0.4 ml) were added to 0.95x10^9^ cells in FA lysis buffer (400 µl). Cells were vortexed (8 cycles, 30 secs, max rpm) and held on ice between cycles (30 secs). A hole was punched through the bottom of the tube with a 30 ½ G needle and the sample spun into a new tube to remove the glass beads. The chromatin sample was pelleted (13,000 rpm, 15 mins, 4°C) and washed with FA lysis buffer (400 µl, 13,000 rpm, 15 mins, 4°C) before suspension in 400 µl chromatin digestion buffer (0.01 M Tris-HCl [pH 8.0], 5 mM MgCl_2_, 0.1% Triton-X100). The sample was then treated with SDS (0.1% final v/v, 37°C, 15 mins) before the addition of 45 µl 11% Triton X-100 (1% final v/v) to remove all unbound SDS.

#### *Chromosome Conformation Capture (3C) Sample Preparation*

Chromatin (52.6 µl) was digested using the restriction enzyme of choice (100 µl final volume, 100 U, 37°C, 2 hrs) before being inactivated by the addition of SDS (1% final v/v) and heat incubation (65°C, 20 mins). Reactions were diluted in T4 ligation buffer (NEB, 2ml) containing Triton X-100 (1% final v/v). T4 DNA Ligase (20 U, Invitrogen) was added and the reaction incubated (16°C, 2 hrs). Samples were reverse cross-linked (65°C, O/N) in the presence of Proteinase K (3.5-5.5 ugml^-1^, Roche), 20 μl EDTA (0.5 M), 12 μl NaCl (5M), 1.2 μl Tris-HCl (1M). RNase A (20 μg) was added and incubated (37°C, 15 mins) prior to three Phenol:Chloroform (1:1) extractions. DNA was precipitated by addition of absolute ethanol (1 ml) with Na Acetate (40 μl, 3 M) and LPA (0.25% final v/v) and incubation (-20 °C, O/N). DNA was pelleted (13,000 rpm, 25 mins, 4°C) and washed with 70% ethanol (700 μl) before suspension in ddH_2_O (40 μl).

#### *Genome Conformation Capture (GCC)*

*Saccharomyces cerevisiae* (BY4741 [Mat**a** his3Δ1 leu2Δ0 met15Δ0 ura3Δ0]) was cultured (30^o^C, 160rpm) to an A_600_ = 0.6 in synthetic complete medium containing amino acid supplements and glucose (2% w/v), glycerol lactate (glycerol 1% v/v, lactate 2% v/v), or galactose (2% w/v). Chromatin was prepared using a modified 3C protocol. Briefly, cells were cross-linked (2% v/v formaldehyde, 10 mins, room temperature, with stirring) and quenched (0.125M glycine). Fifteen samples (residuals of 10^8^ cells in 52.6 μl) were digested (37°C, 2 hrs) with *Msp*I (100 U, Fermentas) in a final volume of 100 µl. *Msp*I was inactivated by the addition of 1% SDS (final v/v) and incubation (65°C, 20 mins). Reactions were diluted in 2 ml T4 ligation buffer (NEB) containing 1% Triton X-100 and external controls added (see below) and T4 DNA Ligase (20 U, Invitrogen) was added and the reaction incubated (16°C, 2 hrs). Samples were reverse cross-linked (65°C, O/N) in the presence of Proteinase K (7-11 ug, Roche), 20 μl EDTA (0.5 M), 12 μl NaCl (5 M), 1.2 μl Tris-HCl (1 M). The second external control (pUC19) and RNase (20 μg) were added and incubated (37°C, 15 mins) prior to three Phenol:Chloroform (1:1) extractions. Samples were purified through 8 columns (Zymo Clean and Concentrator) and each eluted in 15 μl milliQ H_2_O. Samples were vacuum concentrated to a volume of 50 μl. DNA (5 μg in 100 μl) was sequenced using the Illumina Solexa platform (Friedrich Miescher Institute, Basel, Switzerland).

***Saccharomyces cerevisiae reference genome used in the SOAP Alignment***

The reference genome consists of the 16 *S. cerevisiae* nuclear chromosomes (NC_001133.7, NC_001134.7, NC_001135.4, NC_001136.8, NC_001137.2, NC_001138.4, NC_001139.8, NC_001140.5, NC_001141.1, NC_001142.7, NC_001143.7, NC_001144.4, NC_001145.2, NC_001146.6, NC_001147.5, NC_001148.3) as well as the mitochondrial genome (NC_001224.1) and the 2-micron plasmid (YSCPLASM), pUC19 and the ligation control sequences.

***Quantitative 3C Analyses***

Quantitative 3C was performed using Taqman PCRs to analyze the interaction frequency between two *MspI* fragments (nDNA-nDNA, interaction between loci located on Chromosomes VII (bp 868673-873686) and IX (bp 172565 – 173311); Mito-nDNA, interaction between loci located on the Mitochondrial genome (bp 24872 – 26193) and Chromosome XVI (bp 365496-365760)) which were identified by GCC analysis (for primer and fluorescent probe sequences see Table S2).

***Production of external controls for random ligation events***

External controls were produced by the PCR amplification of short regions from the *E. coli* and Lambda phage genomes. Primers (Table S2) were designed to include an *MspI* site at one end of the final product. PCR products were digested with *MspI* (37°C, 2hrs) prior to column purification (Zymo Clean and Concentrator). Purified, digested PCR products were introduced into the GCC samples at a 1:1 ratio with the nuclear genome (unique region) prior to the ligation step during GCC preparation of the glucose and galactose samples. The *E. coli* fragment was introduced into the glucose samples and resulted in 10 ligation events to MspI restriction fragments including eight regions on the nuclear genome, one from the YSCPLASM and one from the mitochondrial genome. The Lambda phage fragment was introduced into the galactose samples and resulted in 50 ligation events with *MspI* fragments including 37 nuclear genome fragments and 13 mitochondrial fragments. No ligation events between external ligation controls and the *S. cerevisiae* genome complement occurred at levels deemed significant.

***Statistical Analyses***

Statistical calculations were performed on datasets in which the sequences could be uniquely positioned on the reference genome. Where the analysis was concerned with connections between elements considered repetitive (*i.e*., the rDNA, mtDNA and 2-micron plasmids) the copy numbers of the elements was corrected for. Connections between unique sequences and connections involving repetitive elements were analyzed separately. Where we analyzed connections between unique loci on the nuclear genome and the mitochondrial genome we considered the mitochondrial genome as unique, as the nuclear genome is the limiting factor. Values for copy number correction were determined from: 1) published reports of the frequencies of these elements within *S. cerevisiae* ; and 2) the frequency of these elements in the GCC sequence files that were returned from the sequencing centres.

During the preparation of the GCC samples random ligation events occur. These events occur during the ligation following restriction enzyme digestion and during the sequencing preparation steps (*i.e*., linker addition during sequencing library preparation). Thus, our null hypothesis is that all interactions we identified are a result of random ligation events, or that there are only random ligations. In an attempt to control for this, two steps were included in our analyses. 1) External controls were added during the GCC library preparation to obtain estimates of the rates of inter-molecular ligation events. 2) We perform statistical analyses to determine whether our GCC dataset is something other than random. To achieve this we performed 100,000 simulations of random pairings (we performed these simulations previously to establish the GCC method, but have repeated them here for these new datasets). Each individual simulation contained the same total number of interactions as the GCC dataset. The maximum count over 100,000 simulations was 6 for the glycerol lactate condition, and 5 for the other two conditions (Table 1). In our real dataset the maximum count we observe for any pairing is 14 for the glucose condition, 32 for galactose and 41 for glycerol lactate (Table 1). Therefore, we conclude that the interaction patterns cannot be attributed to noise alone under any of the conditions, in each case with a p-value less than 10^-5^. Glucose is used as an example below.

***Glucose example for establishing whether the GCC dataset differs from random***

There are 133 mitochondrial segments and 11059 nuclear segments that participate in an interaction; a total of N = 30905 interactions were observed. 100,000 simulations of random pairings, with each segment having equal probability p to pair to another, were performed. In an individual simulation the maximum count for an observed pairing was 5 and only 5 of the 100,000 simulations achieved this value. In our real dataset we observe a maximum count of 14 for an observed pairing. We never observed a maximum count of 14 during our simulations and therefore we conclude that our dataset is non-random with a p-value less than 10^-5^.

Next we wanted to know which of our individual interactions are above experimental noise. It is justified to assume the pairings are independent and therefore the number of times one specific pairing occurs is a binomially distributed random variable. Let S1 and S2 be the number of mitochondrial and nuclear segments, respectively, which participate in at least one interaction. We calculate the probability P(X≥k) where N is number of observed pairings and p is 1 divided by S1 multiplied by S2, for one specific pairing to occur k or more times. L is then S1 multiplied by S2, being the number of possible pairings and we expect to see L∙P(X≥k) pairings occurring k or more times by chance. This provides us with the expected number of false positives. The value k has to be chosen in such a way to provide an acceptable number of false positives and consequently an acceptable false positive rate (see Table 2). k=3 was selected as an acceptable noise cut-off value for each of our samples (Bold).

***GCC copy number determination and statistical analysis of repetitive elements***

Repetitive elements are those genomic features which occur more than once within the genome (*e.g.,* the mitochondrial genome, the rDNA and 2 micron plasmid). We were expressly interested in the interactions between the mitochondrial genome and the nuclear rDNA repeats. We calculated copy numbers for these elements in our samples by aligning the sequence files against sections of these elements (Table S1 and S8).

Mitochondrial copy numbers (Table S1) were calculated for the GCC samples by aligning the complete sequence files against: 1) a unique nuclear element (*GAL1*; Chr II: 279790-279909) and 2) a section of the mitochondrial genome (AI5γ; Mito: 25535-25654; Supplementary Methods). The ratio of mitochondrial sequences to the unique nuclear element was calculated by dividing the percentage of sequence tags that mapped to the AI5γ element by the number of sequences that mapped to the *GAL1* element. Copy number ratios are shown below. This method for empirically calculating mitochondrial copy number makes two key assumptions: 1) that the amount of the AI5γ present in each sample is a true representation of mitochondrial genome copy number; 2) that mitochondrial genome loss is not condition dependent but rather stochastic during sample preparation.

Ribosomal DNA copy numbers (Table 3) were calculated in a similar manner using rDNA (Chr XII: 460517-460612) and *GAL1* (Chr II: 279790-279909) sequences. These calculations resulted in rDNA copy numbers within the expected range .

Interactions between the mitochondrial genome and nuclear rDNA repeats were isolated and subjected to binomial analysis with copy number corrections included (Table 4). The copy numbers for the mitochondria and rDNA were calculated as above, and were integrated into the binomial calculations by altering the number of interacting segments for the probability calculation p (*e.g.* there were 151 mitochondrial *MspI* fragments involved in an interaction, the copy number was found to be 12.19236, thus 151*12.19236 = 1841 mitochondrial fragments). The interaction value cut-off for individual pairings above noise, for each condition, is 3 and is indic*a*ted in Table 4 (bold).

***Sequence Population Statistics***

We wanted to know whether our sequences obtained from the Illumina genome analyser were representative of the *S. cerevisiae* genome. From published reports of copy number we calculated how many base pairs the whole genome complement is and therefore what percentage specific genomic elements should encompass (Table 5). The number of base pairs in the yeast genome complement was calculated by the addition of the 16 yeast chromosomes at a copy number of 1, the rDNA repeats (9.1kb) at a copy number of 200, the mitochondrial genome and 2 micron plasmid at a copy number of 50, for a total of 18,563,326 bp. The sequence files were aligned against the *S. cerevisiae* genome to determine the total number of sequences which could be aligned. We then aligned the sequences against a number of different genomic features to compare the percentage the particular feature should make up against the percentage of sequences which could be aligned against the same feature. The reference genes (housekeeping) were obtained by batch download from the SGD website. The set consisted of 646 verified ORFs across all chromosomes that were between 400-500 amino acids in size. Sequences are available upon request. Table 5 summarises the sequence population statistics.

## Analysis of data published by Duan et al.

The paired end sequences were aligned to the *Saccharomyces cerevisiae* reference genome using SOAP allowing no mismatches and excluding sequences with unidentified nucleotides. The resulting sequence pairs then had to pass a further filtering step: Only if a restriction site of the primary restriction enzyme of the respective experiment lay within 7bp of at least one of the ends of each sequence then the pair was used for further analyses (Data S1-S5).

In order to enable a comparison of our glucose GCC dataset with that of Duan *et al*., both datasets need to use the same genome fragmentation. Given that the *Msp*I fragments are generally smaller than the *Eco*RI and *Hin*dIII genomic fragments, we mapped our data onto an *Eco*RI or *Hin*dIII fragmented genome to minimize boundary effects. The interaction frequencies of *Msp*I fragments that over-lapped *Eco*RI or *Hin*dIII fragments were assigned proportional to the length of the overlap.

Adjacent interactions within the *Msp*I data set were removed prior to mapping onto the *Eco*RI and *Hin*dIII fragments. This avoids the generation of large numbers of false self-ligation products in the re-mapped data. Moreover, it is impossible to determine if adjacent interactions result from a protected restriction site or a digestion and ligation reaction. Therefore, we removed all adjacent interactions from Duan *et al*. and any that resulted from the GCC re-mapping procedure.

False detection rates and noise cut-offs were calculated for the *Eco*RI and *Hin*dIII datasets according to the method outlined for the GCC data. Noise cut-offs are shown in Table S4.

## Blastn comparisons of fragments in interaction pairs.

Genomic sequences for all restriction fragments involved in mito-nDNA interactions within the glucose unique interactions dataset were obtained from the reference genome sequence. The smaller restriction fragment from each interaction pair was compared to the longer fragment by blastn using default parameters. The length, score, and evalue for each comparison was recorded. Comparisons that showed no similarity were given an evalue score of 10. Results are presented in Data S6.

# References

1. Rodley CDM, Bertels F, Jones B, O'Sullivan JM (2009) Global identification of yeast chromosome interactions using Genome conformation capture. Fungal Genetics and Biology 46: 879-886.

2. Cui H, Ghosh SK, Jayaram M (2009) The selfish yeast plasmid uses the nuclear motor Kip1p but not Cin8p for its localization and equal segregation. The Journal of Cell Biology 185: 251-264.

3. Williamson DH, Fennell DJ (1979) Visualization of yeast mitochondrial dna with the fluorescent stain "DAPI". In: Sidney Fleischer LP, editor. Methods in Enzymology: Academic Press. pp. 728-733.

4. Kobayashi T, Heck DJ, Nomura M, Horiuchi T (1998) Expansion and contraction of ribosomal DNA repeats in Saccharomyces cerevisiae: requirement of replication fork blocking (Fob1) protein and the role of RNA polymerase I. Genes & Development 12: 3821-3830.

5. Kobayashi T, Heck DJ, Nomura M, Horiuchi T (1998) Expansion and contraction of ribosomal DNA repeats in Saccharomyces cerevisiae: requirement of replication fork blocking (Fob1) protein and the role of RNA polymerase I. Genes Dev 12: 3821-3830.

6. Duan Z, Andronescu M, Schutz K, McIlwain S, Kim YJ, et al. (2010) A three-dimensional model of the yeast genome. Nature 465: 363-367.

7. Li R, Li Y, Kristiansen K, Wang J (2008) SOAP: short oligonucleotide alignment program. Bioinformatics 24: 713-714.

8. Li R, Li Y, Kristiansen K, Wang J (2008) SOAP: short oligonucleotide alignment program. Bioinformatics 24: 713-714.

# Tables

**Table 1: Empirical and simulated interaction counts for the GCC libraries in this study.**

| **Sample** | **Glucose** | **Glycerol Lactate** | **Galactose** |
| --- | --- | --- | --- |
| **N^o^. Mitochondrial Segments** | 133 | 132 | 131 |
| **N^o^. Nuclear Segments** | 11059 | 12825 | 10748 |
| **N^o^. Interactions Observed** | 30905 | 88107 | 27596 |
| **N^o^. Simulations** | 100000 | 100000 | 100000 |
| **Simulated max count** | 5 | 6 | 5 |
| **N^o^. Simulations Reaching this Count** | 5 | 2 | 2 |
| **Observed max count** | 14 | 41 | 32 |
| **p-value** | < 10^-5^ | < 10^-5^ | < 10^-5^ |

**Table 2: False positive rate calculations for GCC libraries in this study.**

| **Sample** | **k** | **N** | **p** | **P(X≥k)** | **L** | **L∙P(X≥k) Expected number of false positives** | **False Positive Rate** |
| --- | --- | --- | --- | --- | --- | --- | --- |
| **Glucose** | 2 | 30905 | 1/(133*11059) | 2.16E-04 | 1470847 | 317.9225032 | 317.92/1590=0.200 |
| **Glucose** | **3** | **30905** | **1/(133*11059)** | **1.51E-06** | **1470847** | **2.226555129** | **2.23/172=0.012** |
| **Glucose** | 4 | 30905 | 1/(133*11059) | 7.95E-09 | 1470847 | 0.011694801 | 0.012/68=1.76E-4 |
| **Glycerol Lactate** | 2 | 88107 | 1/(132*12825) | 1.29E-03 | 1692900 | 2176.467784 | 2176.47/8884=0.245 |
| **Glycerol Lactate** | **3** | **88107** | **1/(132*12825)** | **2.23E-05** | **1692900** | **37.75726218** | **37.76/1400 = 0.027** |
| **Glycerol Lactate** | 4 | 88107 | 1/(132*12825) | 2.90E-07 | 1692900 | 0.491252849 | 0.49/343=0.001 |
| **Galactose** | 2 | 27596 | 1/(131*10748) | 1.88E-04 | 1407988 | 265.0223343 | 265.02/1384=0.191 |
| **Galactose** | **3** | **27596** | **1/(131*10748)** | **1.23E-06** | **1407988** | **1.732331339** | **1.73/138 = 0.013** |
| **Galactose** | 4 | 27596 | 1/(131*10748) | 6.03E-09 | 1407988 | 0.008487333 | 0.0085/44=1.93E-4 |

**Table 3: Ribosomal DNA copy number calculations**

| Condition | Total No.  sequence reads | No. *GAL1*  aligned reads | % total *GAL1* | No. rDNA  aligned reads | % total  rDNA | Ratio  rDNA:GAL1 |
| --- | --- | --- | --- | --- | --- | --- |
| Glucose | 112335584 | 733 | 0.00065 | 103881 | 0.092 | 141.72 |
| Glycerol Lactate | 98269812 | 767 | 0.00078 | 95001 | 0.097 | 123.86 |
| Galactose | 96838770 | 621 | 0.00064 | 85548 | 0.088 | 137.76 |

**Table 4: Interaction cut-offs and false positive rates for mitochondrial-rDNA interactions analysed in this study.**

| **Sample** | **k** | **N** | **p** | **P(X≥k)** | **L** | **L∙P(X≥k) Expected number of false positives** | **False Positive Rate** |
| --- | --- | --- | --- | --- | --- | --- | --- |
| **Glucose** | 3 | 21358 | 1/(1841*6377) | 1.00E-09 | 11740057 | 0.011763502 | 5.06175E-06 |
| **Glycerol Lactate** | 3 | 33576 | 1/(2425*5574) | 2.54948E-09 | 13516950 | 0.034461207 | 7.49157E-06 |
| **Galactose 2010** | 3 | 11709 | 1/(3135*6199) | 3.6427E-11 | 19433865 | 0.000707917 | 4.52632E-07 |

**Table 5: Population statistics for the sequences used in this study.**

|  | **% of Genome (18,563,326)^a^** | **% of Glucose Sequences (101,767,324)^†^** | **% of Glycerol Lactate Sequences (89,904,522) ^†^** | **% of Galactose Sequences (88,316,472) ^†^** |
| --- | --- | --- | --- | --- |
| **CEN** | 0.010 | 0.004 | 0.005 | 0.004 |
| **TEL** | 0.758 | 1.319 | 1.379 | 1.345 |
| **rDNA 200** | 9.804 | 15.943 | 15.261 | 13.535 |
| **Housekeeping** | 4.755 | 6.702 | 6.800 | 7.015 |
| **YSCPLASM (50)** | 1.702 | 2.428 | 2.256 | 2.274 |
| **Mitochondria (50)** | 23.104 | 3.207 | 5.791 | 5.299 |

^a^ The size of the *S. cerevisiae* genome in base pairs including 200 rDNA repeats, 50 mitochondria, and 50 2 micron plasmids. **^†^** The subset of sequences which could be positioned on the *S. cerevisiae* genome with no mismatches. Chastity filtered sequence files for each condition (glucose, glycerol lactate and galactose) were aligned against the *S. cerevisiae* genome, using the alignment algorithm SOAP to obtain the total number of sequences which could be aligned. The sequence files were then aligned against genomic features to ascertain whether the sequences were a good representation of the *S. cerevisiae* genome. Using published reports of copy number and the reference genome we calculated the likely percentage these features contribute to the total genome complement. We then determined what percentage these features amounted to in the sequence files. Only the centromeric and mitochondrial sequences were lower than expected.
